# Prediction and analysis of essential genes using the enrichments of gene ontology and KEGG pathways

**DOI:** 10.1371/journal.pone.0184129

**Published:** 2017-09-05

**Authors:** Lei Chen, Yu-Hang Zhang, ShaoPeng Wang, YunHua Zhang, Tao Huang, Yu-Dong Cai

**Affiliations:** 1 School of Life Sciences, Shanghai University, Shanghai, People’s Republic of China; 2 College of Information Engineering, Shanghai Maritime University, Shanghai, People’s Republic of China; 3 Institute of Health Sciences, Shanghai Institutes for Biological Sciences, Chinese Academy of Sciences, Shanghai, People’s Republic of China; 4 Anhui province key lab of farmland ecological conversation and pollution prevention, School of Resources and Environment, Anhui Agricultural University, Hefei, People’s Republic of China; Harbin Institute of Technology Shenzhen Graduate School, CHINA

## Abstract

Identifying essential genes in a given organism is important for research on their fundamental roles in organism survival. Furthermore, if possible, uncovering the links between core functions or pathways with these essential genes will further help us obtain deep insight into the key roles of these genes. In this study, we investigated the essential and non-essential genes reported in a previous study and extracted gene ontology (GO) terms and biological pathways that are important for the determination of essential genes. Through the enrichment theory of GO and KEGG pathways, we encoded each essential/non-essential gene into a vector in which each component represented the relationship between the gene and one GO term or KEGG pathway. To analyze these relationships, the maximum relevance minimum redundancy (mRMR) was adopted. Then, the incremental feature selection (IFS) and support vector machine (SVM) were employed to extract important GO terms and KEGG pathways. A prediction model was built simultaneously using the extracted GO terms and KEGG pathways, which yielded nearly perfect performance, with a Matthews correlation coefficient of 0.951, for distinguishing essential and non-essential genes. To fully investigate the key factors influencing the fundamental roles of essential genes, the 21 most important GO terms and three KEGG pathways were analyzed in detail. In addition, several genes was provided in this study, which were predicted to be essential genes by our prediction model. We suggest that this study provides more functional and pathway information on the essential genes and provides a new way to investigate related problems.

## 1. Introduction

As is known, genes are the basic molecular unit of heredity. However, the functions of genes have been widely reported to be redundant and reduplicative [1, 2]. Some genes have turned out to be significant for survival, while others seem to be not necessary. To distinguish these two groups of genes and identify the core heretical regulatory factors, a new concept, named essential genes, has been presented. Essential genes refer to a group of fundamental genes necessary for a specific organism to survive in a specific environment [3]. Based on two reliable and widely quoted literatures, essential genes refer to sets of genes that are absolutely required for indispensable for the viability of individual human cell types [4, 5]. Generally, the essential genes encode conservative functional elements, which mainly contribute to DNA replication, gene translation, gene transcription and substance transportation [6]. Compared to other genes, essential genes convey fewer selective advantages and may have decreased fitness, escaping from the natural selection.

Considering the fundamental role of essential genes that participate in organism survival, for centuries, people have concentrated on the identification of essential genes in various organisms. Bacteria are a perfect experimental model for the identification of essential genes [6, 7]. For bacteria, there are mainly two methods that may contribute to the identification of essential genes in the genome-scale: gene direct deletion and transposon-based randomized mutagenesis [8, 9]. Relying on two such functional experimental methods, the essential and non-essential genes in various microorganism species have been identified and distinguished from each other. The well-known pathogen *Helicobacter pylori* is an example. *H*. *pylori* is a gram-negative pathogen that is usually found in the stomach [10]. Based on whole population mutagenesis and microarray analysis, there have been 1,576 candidate ORFs identified in the genome of *H*. *pylori*. After screening, there were 1,178 ORFs that were non-essential genes, which left approximately 25% essential genes. However, such screening processes have mostly been applied in prokaryotic organisms and yeast. It is difficult to do similar screenings in multicellular organisms, especially in mammals.

With the development of screening technologies, the essential genes in mice have been generally revealed [11]. Based on microinject KO and nuclear transfer techniques, such research has identified that at least 2,114 genes are essential in mice [12]. However, due to ethical arguments, such an experimental method cannot be repeated in humans. Traditionally, the identification of human essential genes mainly depends on literature searching, which is not comprehensive and is generally biased [13]. In 2016, following the rapid development of genome-editing techniques, especially the CRISPR/Cas9 genome editing system, a further trial on essential gene screening in human cells was reported [14]. In the study, the research group compared the editing efficacy of traditional RNAi techniques and the newly applied CRIPSR/Cas9 system, and further identified a group of experimental validated essential genes and non-essential genes in the human chronic myelogenous leukemia cell line K562, providing a new experimental tool for essential gene identification [14]. Although, in such a study, large series of essential genes have been revealed, it is not the ultimate goal for us to identify essential genes. Actually, the fundamental biological processes the directly affect the physical and pathological processes in human bodies was our focus. However, until now, how essential genes systematically contribute to fundamental biological processes has not been fully revealed and analyzed in detail. Among the computational methods for predicting essential genes, the first one was provided in 1996 by Mushegian and Koonin to identify a minimal set of essential genes from 468 protein-encoding genes in *Mycoplasma genitalium*. Finally, 256 genes were selected and regarded as the possible minimal gene set to maintain the existence of modern-type cells [15]. In prediction methods using machine learning algorithms, some that are commonly used, including support vector machine (SVM), neural networks, Naïve Bayes, and decision trees, were used to predict essential genes based on biological and topological features. Detailed information on this can be found in the excellent review [16]. Specifically, Zhong *et al*. provided a gene expression programming-based (GEP) method to predict essential genes in *S*. *cerevisiae* using a combination of features. The constructed classifier was comprehensively evaluated by eight measurements. The results showed that the GEP classifier outperformed other methods using individual features and received a better AUC score than most of the classifiers trained by various machine learning algorithms [17].

In the field of bioinformatics, several computational methods have been proposed to tackle various problems [18–20]. Among them, the biological sequence analysis methods were always used to investigate the DNA-related and protein-related problems [21–24]. On the other hand, the effective clustering of functional genes, mainly based on gene ontology (GO) and KEGG pathways [25, 26], which cluster functional genes into different biological processes, is also useful to tackle these problems. In this study, we investigated the essential and non-essential genes using GO terms and KEGG pathways. Unlike the computational methods mentioned above, we aimed to identify important GO terms and KEGG pathways that can be important indicators for distinguishing essential and non-essential genes, and at the same time a classification model was built using these GO terms and KEGG pathways. The essential/non-essential genes of the human chronic myelogenous leukemia cell line K562 were retrieved from Morgens *et al*.’s study [14]. The relationship between one gene and one GO term or KEGG pathway was encoded into a numeric value using the enrichment theory of GO and KEGG. Then, some popular computational methods, maximum relevance minimum redundancy (mRMR) [27], incremental feature selection (IFS), and a support vector machine (SVM) [28, 29], were employed to analyze involved GO terms and KEGG pathways. As a result, some key GO terms and KEGG pathways were extracted. Among them, the most important ones were extensively analyzed. In addition, a prediction model was proposed to distinguish essential and non-essential genes, which provided nearly perfect performance, with a Matthews correlation coefficient of 0.951. In consideration of the perfect performance of the prediction model, it was applied to identify essential genes from unlabeled ones, yielding several possible essential genes. Considering the crucial role of essential genes investigated in this study, the biological processes described by our identified GO terms and KEGG pathways may be fundamental for cell survival. Therefore, for the first time, we screened out and analyzed the essential genes at functional levels.

## 2. Materials and methods

### 2.1 Dataset

We used the gold standard 217 essential genes and 927 non-essential genes that were compiled by Morgens *et al*. [14]. These essential genes were critical for cell growth [30] and are provided in S1 Table. For convenience, essential genes were deemed positive samples, while non-essential genes were regarded as negative samples. The purpose of this study was to explore the functional difference between essential genes and non-essential genes. In detail, we aimed to extract important GO terms and biological pathways that can perfectly discriminate essential and non-essential genes. In view of this, twelve essential genes and 97 non-essential genes were discarded because their enrichment scores on GO terms and biological pathways were not available; i.e., 205 essential genes and 830 non-essential genes were investigated in this study.

### 2.2 Representation of essential and non-essential genes

To extract important GO terms and pathways that are tightly related to essential genes, all genes, including essential and non-essential genes, should be encoded by GO terms and pathways. Then they can be analyzed by various computational methods. In this study, we used the enrichment theory [31] of GO terms and KEGG pathways, which can encode the relationship between one gene and one GO term (KEGG pathway) into a numeric value. Its brief description is as follows.

#### 2.2.1 GO enrichment score

For a given gene *g* and one GO term GO_*j*_, let *G*_*GO*_ denote a gene set consisting of annotated genes of GO_*j*_ and *G*(*g*) denotes another gene set consisting of *g* and its direct neighboring genes in the protein-protein interaction network reported in STRING. The GO enrichment score of *g* and GO_*j*_ is defined as the hypergeometric test *P* value [32–37] on *G*(*g*) and *G*_*GO*_, which can be computed by:

$$S_{\text{GO}}\text{(}g\text{,}GO_{j}\text{)}=-{\text{log}}_{10}(\overset{n}{\underset{k=m}{\sum }}\frac{\left(\begin{array}{c} M\\ k \end{array}\right)\;\left(\begin{array}{c} N-M\\ n-k \end{array}\right)}{\left(\begin{array}{c} N\\ n \end{array}\right)})$$ (1)

where *N* and *M* denote the total number of human genes and the number of genes in *G*_*GO*_; *n* and *m* represent the number of genes in *G*(*g*) and the number of genes in both *G*(*g*) and *G*_*GO*_. The higher the score is, the stronger the functional association of the gene *g* and GO term GO_*j*_. A total of 17,916 GO terms were used in this study, inducing 17,916 GO enrichment scores.

#### 2.2.2 KEGG enrichment score

A similar method can be applied to obtain the KEGG enrichment score of one gene and one pathway, which can measure the relationship between them. Let *G*_*KEGG*_ denote a set consisting of annotating genes of one KEGG pathway *K*_*j*_. The KEGG enrichment score of *g* and *K*_*j*_ is defined to be the hypergeometric test *P* value [32–37] on *G*(*g*) and *G*_*KEGG*_, which can be calculated by:

$$S_{\text{KEGG}}\text{(}g\text{,}K_{j}\text{)}=-{\text{log}}_{10}(\overset{n}{\underset{k=m}{\sum }}\frac{\left(\begin{array}{c} M\\ k \end{array}\right)\;\left(\begin{array}{c} N-M\\ n-k \end{array}\right)}{\left(\begin{array}{c} N\\ n \end{array}\right)})$$ (2)

where *N* and *n* are same as those in Eq 1, *M* denotes the number of genes in *G*_*KEGG*_, and *m* denotes the number of genes in both *G*(*g*) and *G*_*KEGG*_. Similarly, a large KEGG enrichment score means the strong functional association between the gene *g* and the KEGG pathway *K*_*j*_. A total of 279 KEGG pathways were used in this study, resulting in 279 KEGG enrichment scores.

Each essential or non-essential gene can be represented by 17,916 GO enrichment scores and 279 KEGG enrichment scores. In other words, each gene *g* can be encoded into an 18,195-D vector, formulated as:

$$v(g)={\left[S_{GO}(g,GO_{1}),\cdots ,S_{GO}(g,GO_{17916}),S_{KEGG}(g,K_{1}),\cdots ,S_{KEGG}(g,K_{279})\right]}^{\text{T}}$$ (3)

### 2.3 Feature evaluation using the mRMR method

As mentioned in Section 2.2, several GO terms and KEGG pathways were employed to represent essential genes and non-essential genes. However, not all of them can provide positive contributions for discriminating them. To analyze them, a reliable and widely used feature selection method; i.e., the mRMR method [27], was employed in this study. This method has been widely applied to analyze several complicated biological problems [35, 37–44]. Two excellent criteria were used in this method: maximum relevance and minimum redundancy. Each feature was evaluated from two aspects: (1) the relevance to the target and (2) the redundancy to other features. Based on them, a feature list, namely, the mRMR feature list, can be obtained in which features are ranked in a rigorous way. The description of how to obtain this list is described below.

The mRMR method is a mutual information (MI)-based feature selection method, and all evaluations are based on the MI of two variables *x* and *y*, which can be computed according to the following equation:

$$I(x,y)=\iint p(x,y)\text{log}\frac{p(x,y)}{p(x)p(y)}dxdy$$ (4)

where *p*(*x*, *y*) is joint probabilistic density of *x* and *y*, *p*(*x*) and *p*(*y*) are their marginal probabilistic density. A large MI value between *x* and *y* indicates the strong association between them. For a dataset in which each sample is represented by *N* features, let Ω be a set consisting of *N* features, Ω_*s*_ be a set consisting of the already selected features (initially, it is set to an empty set), Ω_*t*_ be a set containing the rest features. In the next round, a feature in Ω_*t*_ is selected and moved to Ω_*s*_. For each feature *f* in Ω_*t*_, we computed its relevance to target *c* by

D=I(f,c) (5)

In addition, its redundancy to features in Ω_*s*_ is also considered, which can be evaluated by:

$$R=\frac{1}{\left|\Omega_{s}\right|}\underset{f_{i}\in \Omega_{s}}{\sum }I(f,f_{i})$$ (6)

In particular, if Ω_*s*_ is an empty set, *R* will be set to zero. To integrate the relevance to the target and the redundancy to already selected features, we further compute *D*-*R* for each feature in Ω_*t*_. The feature with the maximum *D*-*R* value can be found and it is removed from Ω_*t*_ to Ω_*s*_. When all features are in Ω_*s*_, the whole procedures stop. All features are ranked according to their selection orders, producing the mRMR feature list, formulated as

$$F=[f_{1},f_{2},\cdots ,f_{N}]$$ (7)

In addition to the mRMR feature list, the mRMR method also yields another feature list, MaxRel feature list, in which features are ranked by the decreasing order of their MI values to targets. It is clear that features receiving high ranks in this list can give key contributions for classification.

### 2.4 IFS method

The mRMR method ranks all features in the mRMR feature list only. However, which features should be selected to participate in classification is still a problem. Here, the IFS method was employed. Based on the mRMR feature list, *F*, we can construct a series of feature sets. Each set contained some top features in the list *F*. For each constructed set, say *F*′, all essential and non-essential genes were represented by features in *F*′. Then, a classification algorithm was executed on these genes, with its performance evaluated by ten-fold cross-validation [45]. By testing all possible feature sets or some of them, the feature set yielding the best performance can be obtained. This feature set was called the optimal feature set and features in this set were named optimal features. In addition, a prediction model can be constructed using the optimal features to represent genes and the classification algorithm as the prediction engine.

### 2.5 SVM algorithm

According to the description of the IFS method in Section 2.4, one classification algorithm is necessary. Here, we selected one of the classic machine learning algorithms, the SVM algorithm [28, 29]. The model based on this algorithm can be constructed on a dataset with small size, whereas it can provide good generalization performances. Thus, this algorithm has been widely used in bioinformatics [46–49]. In the algorithm, samples in the dataset are mapped into a higher-dimensional space using the kernel trick, in which the set of positive and negative samples can easily be separated by a hyper-plane with maximum margin. For a query sample, it is also mapped to the same higher dimension and its predicted class depends on which side of the hyper-plane it falls on.

In this study, a type of SVM algorithm was adopted, which is optimized by the sequential minimum optimization (SMO) algorithm [50] proposed by Platt. Different from the traditional optimization methods, the SMO method partitions the original quadratic programming (QP) problem into several smallest sub-QP problems and solves them in an analytical way. To quickly implement this type of SVM, we employed the tool, namely, SMO, in Weka [51], a suite of software collecting several popular machine learning algorithms. For convenience, it was executed using its default parameters.

### 2.6 Accuracy measurements

Because one gene is either an essential or a non-essential gene, it is a binary classification problem. For the predicted results of a binary classification problem, they can be counted as true positive (TP), true negative (TN), false positive (FP), and false negative (FN). In detail, TP/TN represents the number of positive/negative samples that are predicted correctly, and FP/FN represents the number of negative/positive samples that are predicted incorrectly. In addition, these values can induce four measurements: sensitivity (SN), specificity (SP), accuracy (ACC) [52], and Matthews correlation coefficient (MCC) [53], which can be calculated by:

$$SN=\frac{TP}{TP+FN}$$ (8)

$$SP=\frac{TN}{TN+FP}$$ (9)

$$ACC=\frac{TP+TN}{TP+TN+FP+FN}$$ (10)

$$MCC=\frac{TP\times TN-FP\times FN}{\sqrt{\left(TP+FP)(TP+FN)(TN+FP)(TN+FN\right)}}$$ (11)

It is easy to see that SN and SP represent the prediction accuracy of positive and negative samples, respectively. Thus, they are not proper to evaluate the predicted results on the whole. Because the non-essential genes are more than four times as many as essential genes, ACC is also inappropriate. MCC is more proper because it is a balanced measurement even if the sizes of positive and negative samples have a great difference. Furthermore, it has been used as the major measurement in several studies [35, 37, 54–57]. Thus, it was selected as the major method to evaluate the predicted results yielded by different prediction models.

## 3. Results and discussion

**Fig 1:**
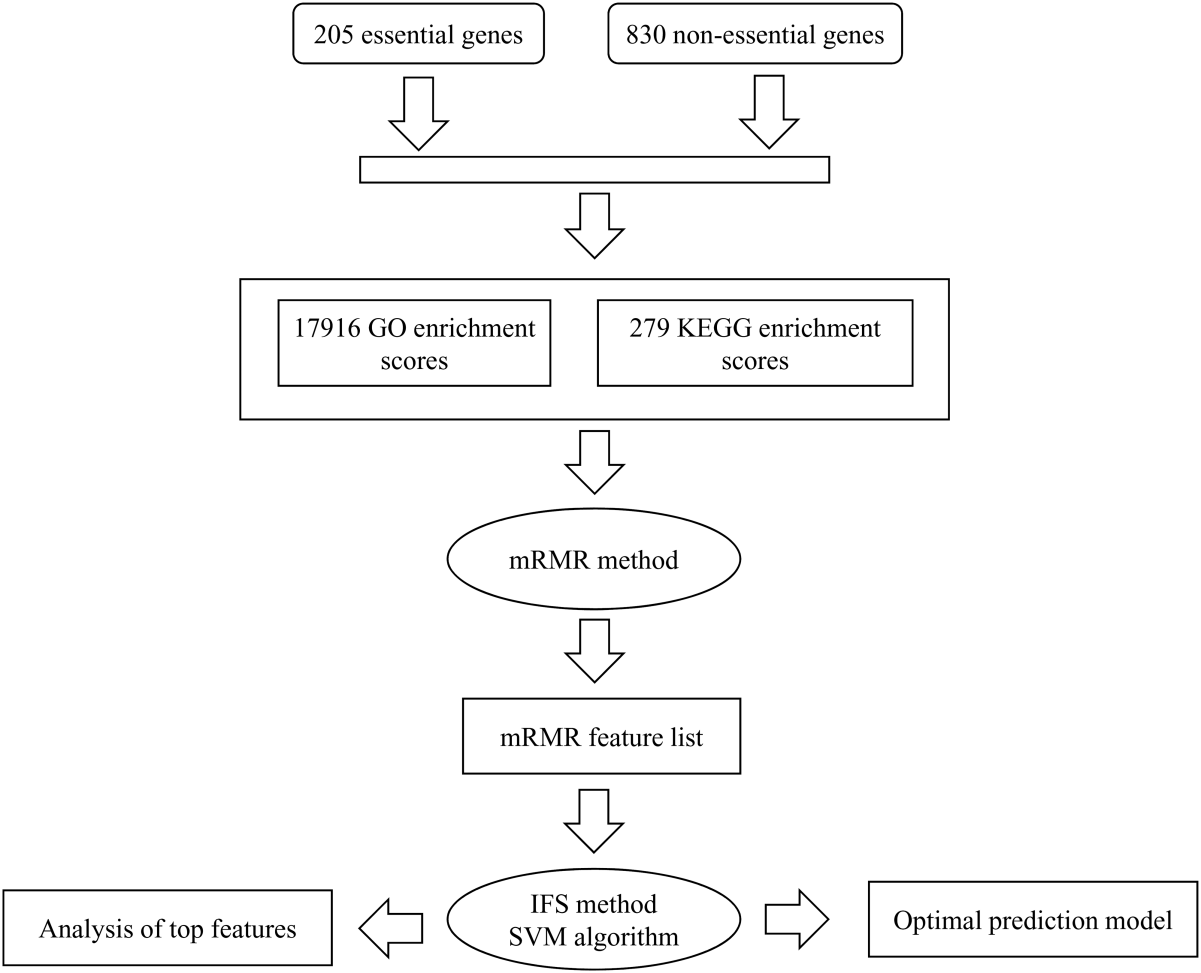
Flow chart of the whole procedure for investigating essential and non-essential genes.

In this study, we used some computer techniques, such as the mRMR method, IFS method and SVM algorithm, to extract important GO terms or KEGG pathways that can discriminate essential genes and non-essential gene to the extent possible. The whole procedures are illustrated in Fig 1. This section gives detailed results of each procedure and the analysis of the identified GO terms or KEGG pathways.

### 3.1 Results of the mRMR method

As mentioned in Section 2.2, each essential gene and non-essential gene was represented by several features that were extracted from the GO terms and KEGG pathways. To analyze them, the mRMR method was employed, resulting in an mRMR feature list *F* and a MaxRel feature list, in which all features were ranked in a rigorous way. The obtained mRMR feature list and MaxRel feature list are provided in S2 and S3 Tables, respectively.

### 3.2 Results of the IFS method

**Fig 2:**
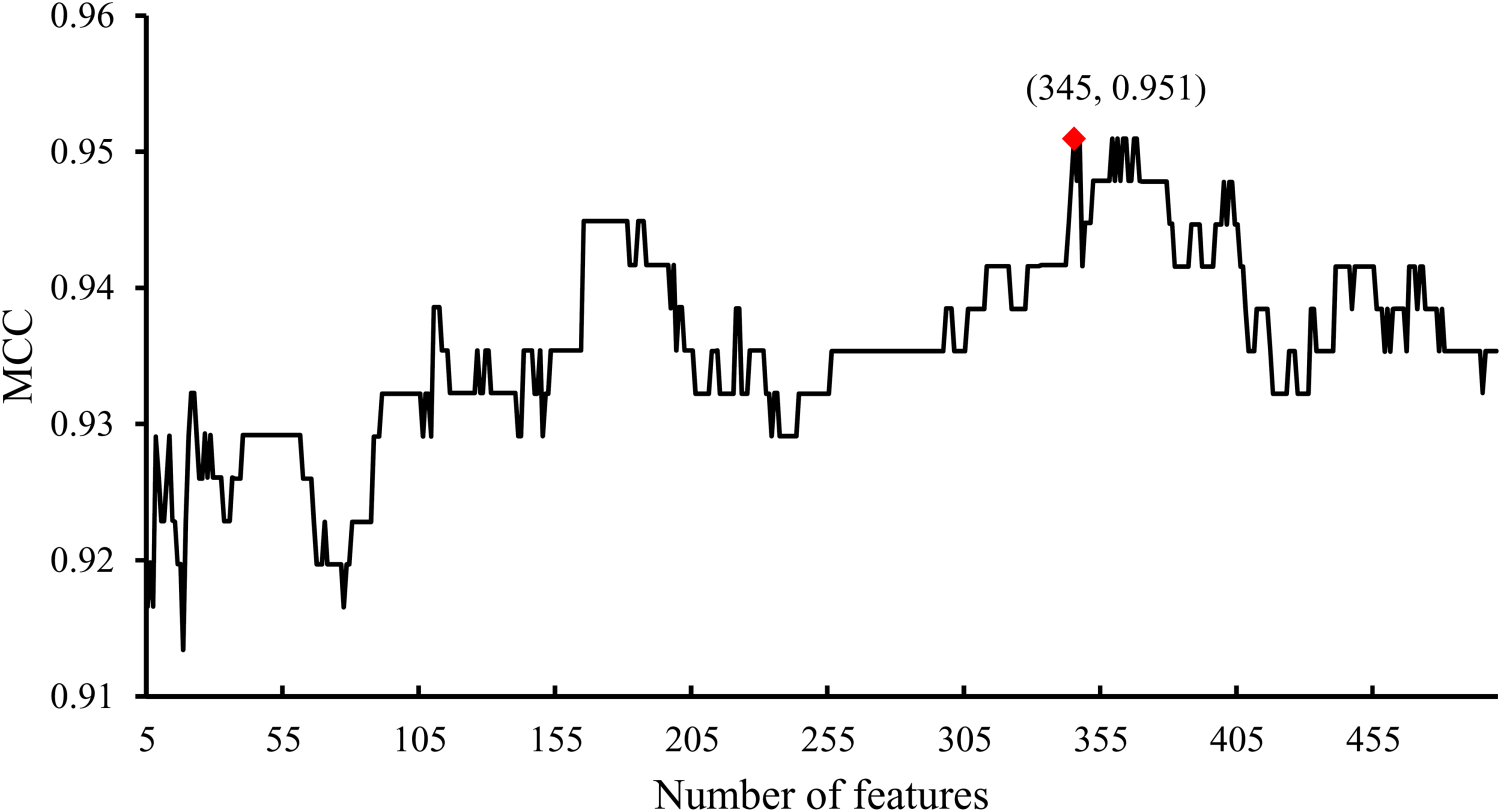
The IFS curve using the MCC as its Y-axis and the number of features participating in classification as its X-axis.

To extract the optimal combination of features that can provide key contributions for discriminating essential and non-essential genes, the IFS method was adopted as mentioned in Section 2.4. However, if all possible feature sets were tested, it would take a lot of time because there were 18,195 features totally. On the other hand, it is impossible that several GO terms and KEGG pathways can indicate the differences between essential and non-essential genes. Thus, we tried feature sets containing features from 5 to 500; i.e., feature sets *F*_*i*_ = {*f*_1_,*f*_2_,⋯,*f*_*i*_} (5≤*i*≤500) were tested. For each feature set, we adopted the SVM algorithm, evaluated by ten-fold cross-validation, to examine it, inducing the measurements SN, SP, ACC, and MCC. After testing these 496 feature sets, a series of SNs, SPs, ACCs and MCCs were obtained, which are available in S4 Table. Because MCC was selected as the key measurement, we found a feature set yielding the maximum MCC. To give a clear observation, we plotted a curve, namely, an IFS-curve, which used the MCC as its Y-axis and the number of features participating in classification as its X-axis, as shown in Fig 2. It can be observed that this curve generally follows an increasing trend in the beginning. It is reasonable because increasingly important GO terms and KEGG pathways participated in the classification procedure. The maximum MCC was 0.951 when the first 345 features in the mRMR feature list were used. Thus, the feature set containing these features was called the optimal feature set and these 345 features were termed as optimal features. Furthermore, an optimal prediction model can be constructed using the optimal features to represent genes and SVM as the prediction engine. The detailed performance of this model is shown in Table 1, from which we can see that the SN, SP and ACC were 0.927, 0.999 and 0.985, respectively, indicating that the model is almost a perfect prediction model.

**Table 1:** The *SN*, *SP*, *ACC*, and *MCC* yielded by the optimal SVM prediction model and the model using features of KEGG enrichment scores.

Model	Number of features	SN	SP	ACC	MCC
Optimal SVM prediction model	345	0.927	0.999	0.985	0.951
Model using features of KEGG enrichment scores	279	0.873	0.989	0.966	0.891

**Fig 3:**
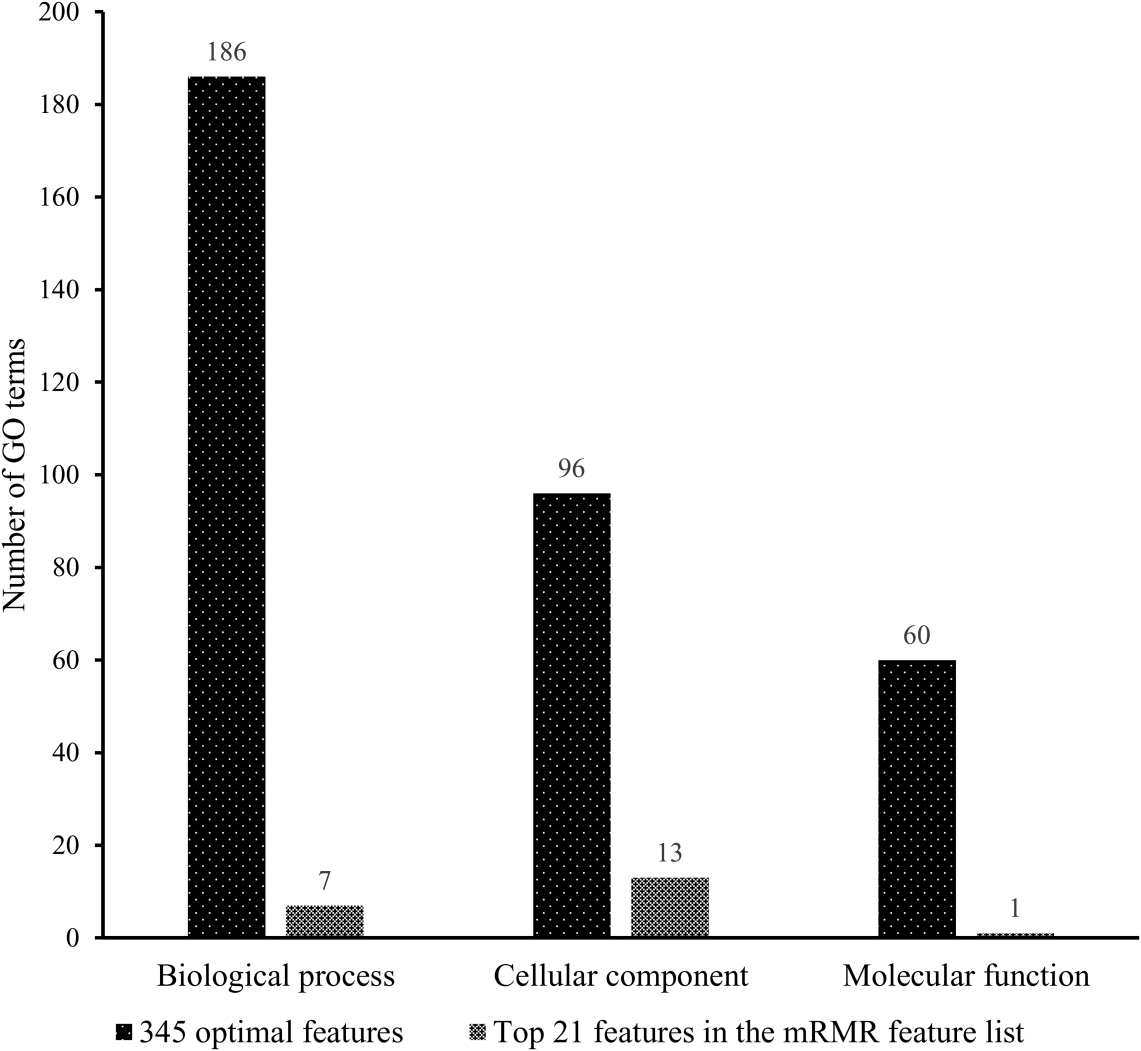
Distribution of the corresponding GO terms of the optimal features and top 21 features in the mRMR features in the three groups.

As mentioned above, 345 top features in the mRMR feature list were used to construct the optimal prediction model. Among these 345 features, 342 were derived from 342 GO terms and three were from three KEGG pathways. Three KEGG pathways were hsa03015 (mRNA surveillance pathway), hsa03013 (RNA transport) and hsa03020 (RNA polymerase). For GO terms, it is known that all GO terms can be clustered into three groups: (1) Biological process (BP); (2) Cellular component (CC) and (3) Molecular function (MF). The distribution of 342 GO terms on these three groups is illustrated in Fig 3, from which we can see that BP GO terms were most, followed by CC GO terms and MF GO terms.

### 3.3 Analysis of important GO terms

**Fig 4:**
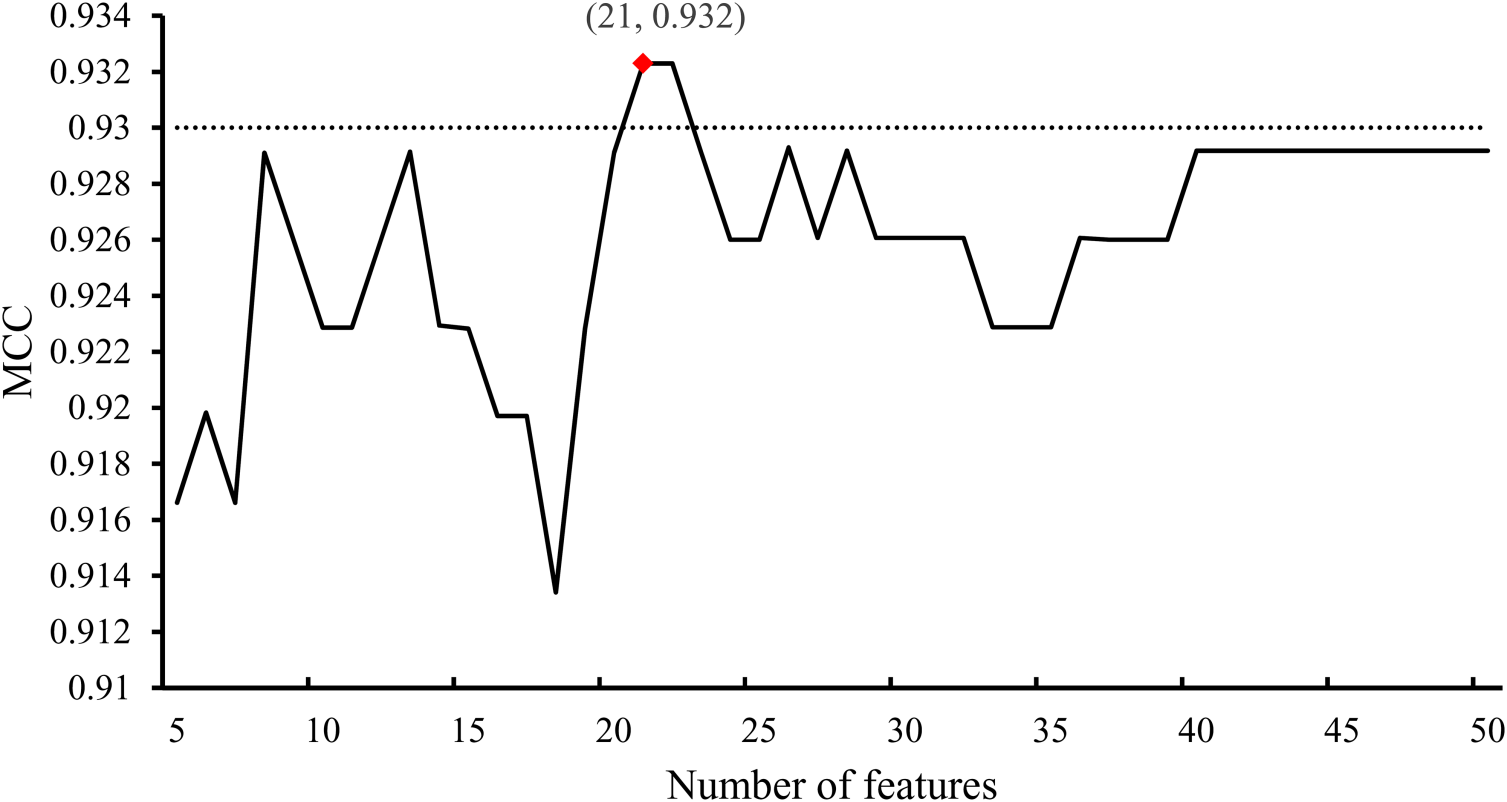
A part of the IFS curve shown in Fig 2.

A total of 345 optimal features were extracted using the IFS method and SVM algorithm. However, it is difficult to analyze all of these 345 features one by one. It is easy to know that features with higher ranks in the mRMR features are more important. Thus, we tried to analyze only some top features in the mRMR features. To determine these features, we amplified the IFS curve between X-axis 5 and 50, which is shown in Fig 4. It can be seen that the MCC value first exceeds 0.930 when the first 21 features in the mRMR feature list were used. Here, we analyzed the corresponding GO terms or KEGG pathways of these features, which are listed in S5 Table. All 21 features were derived from GO terms, which are listed in Table 2. Like the 342 GO terms mentioned in Section 3.2, the distribution of 21 GO terms on three groups is also illustrated in Fig 3. In detail, seven were BP GO terms, thirteen were CC GO terms and one was an MF GO term.

**Table 2:** Twenty-one GO terms corresponding to the top 21 features in the mRMR feature list.

Rank in mRMR feature list	GO term ID	GO term	Cluster
1	GO:0032991	macromolecular complex	Cellular component
2	GO:0021888	hypothalamus gonadotrophin-releasing hormone neuron development	Biological process
3	GO:0071008	U2-type post-mRNA release spliceosomal complex	Cellular component
4	GO:0044424	intracellular part	Cellular component
5	GO:0000154	rRNA modification	Biological process
6	GO:0043226	organelle	Cellular component
7	GO:0016071	mRNA metabolic process	Biological process
8	GO:0071146	SMAD3-SMAD4 protein complex	Cellular component
9	GO:0072669	tRNA-splicing ligase complex	Cellular component
10	GO:0044422	organelle part	Cellular component
11	GO:0021886	hypothalamus gonadotrophin-releasing hormone neuron differentiation	Biological process
12	GO:0002183	cytoplasmic translational initiation	Biological process
13	GO:0005622	intracellular	Cellular component
14	GO:0015030	Cajal body	Cellular component
15	GO:0030874	nucleolar chromatin	Cellular component
16	GO:0044446	intracellular organelle part	Cellular component
17	GO:0010467	gene expression	Biological process
18	GO:0043227	membrane-bounded organelle	Cellular component
19	GO:1902369	negative regulation of RNA catabolic process	Biological process
20	GO:0044822	poly(A) RNA binding	Molecular function
21	GO:0005737	cytoplasm	Cellular component

Among the 342 GO terms which derived the features in the optimal feature set, 186 GO terms turn out to describe biological processes, 60 turn out to describe molecular functions and 96 turn out to describe cellular component (see Fig 3). Therefore, from the overall distribution regardless of the contribution value and degree of correlation, the BP indeed are more informative. However, from the distribution of 21 GO terms shown in Fig 3, CC associated terms are more significant. In fact, CC associated terms are indeed more significant than BP and MF associated terms (with higher ranks in the mRMR feature list). Some literature can support that such fact is true. First, a literature presented by Li *et al*. confirmed that the subcellular location of essential genes is quite significant [58]. Second, considering that most of the essential genes contribute to the maintenance of cell proliferation and survival, it is quite reasonable to predict various subcellular locations as significant factors because most of such proliferation and survival associated biological processes happen in the same place (subcellular locations) and involve specific functional proteins, which are all described by CC, like post-mRNA release spliceosomal complex and SMAD3-SMAD4 protein complex. Therefore, it is reasonable that more CC associated terms were listed in the top of mRMR feature list because one cellular component may involve in multiple biological processes and molecular function that connected to essentialness but in turn are not. In the following text, all 21 GO terms were analyzed.

#### 3.3.1 Analysis of important CC GO terms

Among the 21 important GO terms, there were thirteen CC GO terms. Such CC GO terms have all been confirmed to be related to the survival and proliferation of leukemia cell line K562. **GO: 0044422** (organelle part) describes any constituent part of an organelle including the nucleus, mitochondria, plastids, vacuoles, vesicles, ribosomes and the cytoskeleton, except for the plasma membrane. Considering the constituent part of such cell organelles may definitely involve the survival of the whole cell, genes that can be enriched in this GO term may definitely be fundamental genes, which can be clustered as essential genes [59]. Recent studies on yeast, cancer cells and Neurospora crassa also confirmed the conclusion, circumstantially proving the accuracy and efficacy of GO: 0044422 being a functional benchmark to distinguish essential and non-essential genes [59–61]. Similar with GO: 0044422 describing the organelle part, **GO: 0043226** (organelle) describing the organized structure of organelles may also distinguish essential from non-essential genes in the same way. As the related GO terms of such two CC GO terms we have analyzed above, three specific GO terms, **GO: 0044446** (intracellular organelle part), **GO: 0005622** (intracellular) **and GO: 0005737** (cytoplasm), may also be optimal classifying standards for the identification of essential and non-essential genes. GO: 0044446, which describes the intracellular organelle part of cells, similar to GO: 0044422, has also been confirmed to contribute to the cell survival processes [62]. GO: 0043229 describes all the intracellular organelles, which is also the parental GO term for GO: 0044422 we have analyzed in detail above. For GO: 0005622 and GO: 0005737, these two GO terms describe the general intracellular and cytoplasm cell components, respectively. As we have analyzed above, crucial genes that play irreplaceable roles during the formation and normal metabolism processes of cell organelles may definitely be essential genes [63, 64]. In addition, a specific GO term named **GO: 044424** (intracellular part) has also been identified as a specific GO term that involves essential regulatory processes in our model cell line. Describing any constituent part of the living contents of a cell, including GO: 0044422 and GO: 0044446, as like what we have analyzed above. Considering that these two GO terms have both been confirmed to be essential gene associated GO terms, it is reasonable to regard GO: 044424 as another specific essential gene associated GO term.

Apart from such general CC GO terms, we also obtained some detailed CC GO terms that may be essential for the survival of our target cells, the K562 cells. The crucial GO term **GO: 0043227** (membrane-bounded organelle) may tell the differences between essential genes and non-essential genes. The nucleus, mitochondria, plastids, vacuoles, and vesicles are all fundamental cellular organelles for cell survival, as we have analyzed above [59]. Taking a specific membrane-bounded organelle; i.e., mitochondria, as an example, mitochondria produce the energy currency of the cell and have been confirmed to be essential for the survival of both normal cells and pathological cells, including tumor cells [65, 66]. Therefore, GO: 0043227 may definitely distinguish non-essential from the essential genes. **GO: 0071018** (U2-type post-mRNA release spliceosomal complex) has been screened to be a biological process that could be used to distinguish essential from non-essential genes. As is known, in leukemia cells, the specific mutations of the spliceosome have been confirmed to contribute to the initiation and progression of such malignant diseases [67, 68]. Considering the regulatory role of the spliceosome complex in leukemia cells, the spliceosome has been widely reported to be a candidate therapeutic target of such diseases, implying the essential role of this GO term for leukemia cell survival [67, 69]. Apart from GO: 0071008, another crucial functional complex, **GO: 0032991** (macromolecular complex), was also identified as a crucial judgment factor. The macromolecular complex has been widely reported to contribute to regulation of cell migration and drug resistance in leukemia cells, including K562 cell lines [70, 71]. Therefore, GO term GO:0032991 may definitely contribute to the identification of essential genes. As we have analyzed above, the transcription and translation regulatory role may be essential for the target K562 cells [67, 68]. A specific GO term **GO: 0072669** (tRNA-splicing ligase complex) may also be a crucial reference standard for essential or non-essential genes. As is known, the tRNA-splicing ligase complex contributes to the splicing and formation of tRNA molecules [72]. In leukemia cells, the regulation of functional tRNA molecules may involve various fundamental biological processes, including cell death and morphologic changes, implying that such GO terms may also distinguish essential from non-essential genes [73, 74].

In addition, **GO: 0015030** (Cajal body) was also identified. The Cajal body (CB), which can also be named as coiled bodies, are specific spherical sub-organelles that mainly contribute to the regulation of the telomerase assembly and cell cycle [75, 76]. As we have analyzed above, the cell cycle regulators may definitely be essential for the leukemia cell line K562. Also, a specific GO term **GO: 0030874** (nucleolar chromatin) may also contribute to the distinction of essential and non-essential genes. Nucleolar chromatin turns out to reflect the portion of nuclear chromatin associated with the nucleolus [77, 78]. For leukemia cells, such CC GO terms have been confirmed to regulate the morphological alteration and apoptosis of leukemia cells, validating the essential role of genes contributing to such biological process for leukemia cells [79]. **GO: 0071146** (SMAD3-SMAD4 protein complex) turns out to be a significant indicator for essential genes. According to recent publications, this CC GO term has been validated to contribute to the transforming growth factor-beta signaling pathway, which further participates in the proliferation, survival and transformation of normal and malignant somatic cells, including leukemia cells [80, 81].

#### 3.3.2 Analysis of important BP GO terms

Apart from the CC GO terms, which we analyzed above, we also obtained a group of BP GO terms. Four of the GO terms that can be clustered into RNA associated regulated processes have also been identified as an inspection standard to distinguish essential and non-essential genes. **GO: 0016071** (mRNA metabolic process) is involved in all the chemical reactions and pathways that may involve RNAs. We identified this GO term to distinguish essential genes from non-essential genes. As is known, the expression of certain crucial genes is essential for the initiation and progression of various tumor subtypes including leukemia [82–84]. Since the abnormal expression of certain genes, as we have mentioned above, may definitely initiate the malignant process in leukemia and all the expression biological processes have to rely on the metabolism of RNAs, especially mRNAs, it is reasonable to regard genes contributing to mRNA metabolism as essential genes for the K562 cell line [85–87]. As the reverse processes of such biological processes, **GO: 1902369** (negative regulation of RNA catabolic process) has also been screened out in this study, implying its separating capacity to distinguish essential from non-essential genes. In leukemia cells, recent publications also confirmed that aberrant RNA splicing and editing processes involving these GO terms affect the drug sensitivity of leukemia cells, implying its specific role for cell survival [88]. Apart from that, the catabolic process of RNA has been further validated to regulate starvation- and rapamycin-induced autophagy in K562 cell lines [89]. Similarly, another GO term, **GO: 0000154** (rRNA modification) may also tell the differences between essential and non-essential genes. As is known, the rRNAs contribute to the formation and normal function of ribosomes [90–92]. As the downstream of the transcription processes analyzed above, rRNA associated translation processes are also essential and fundamental for the normal expression of every single protein, not to speak of further biological functions such as cell survival [92, 93]. Further, in leukemia cells, rRNA associated modification has been confirmed to contribute to the elicitation of growth arrest, validating the essential role of this GO term for cell survival [94, 95]. Further, we identified a more detailed biological process that may distinguish essential from non-essential genes, **GO: 0002183** (cytoplasmic translational initiation). This GO term includes the first two amino acids of a protein and the formation of a complex of the ribosome, mRNA, and an initiation complex [96–98]. It has been confirmed that the first two amino acids of a protein are significant for the normal translation of such a protein [99]. Consider a specific gene, SNAT2, as an example. As the neutral amino acid transporter, the depletion of such a gene may be fatal for cells, including leukemia cells, validating the essential role of genes clustered in such GO terms [99, 100]. Therefore, our screened GO term may definitely be essential for the translation of all the candidate mRNAs, implying the essential role of genes that can be clustered in such GO terms for further cell survival.

Further, we also obtained a parental GO term of the GO terms that we analyzed above; i.e., **GO: 0010467** (gene expression). According to the analysis above, the expression processes of certain genes may definitely be essential for the survival of human cells, including the K562 leukemia cell line [87, 94, 101]. Therefore, all BP GO terms have been confirmed to distinguish essential genes from non-essential genes based on recent publications. We also obtained two specific hypothalamus gonadotrophin-releasing hormone neuron associated biological processes; i.e., **GO: 0021886** (hypothalamus gonadotrophin-releasing hormone neuron differentiation) and **GO: 0021888** (hypothalamus gonadotrophin-releasing hormone neuron development). As is known, as a functional component of the neuroendocrine regulation of reproductive function, the hypothalamus gonadotrophin-releasing hormone has been confirmed to contribute to the survival and proliferation of leukemia cells by interacting with leukemia inhibitory factor and interleukin-6 [102, 103].

#### 3.3.3 Analysis of important MF GO terms

As for molecular functions, only one GO term, named **GO: 0044822** (poly(A) RNA binding), contributes to the poly (A) RNA binding process. Generally, such poly (A) binding processes refer to the regulation of transcription and translation of certain genes. In leukemia, it is significant for the regulation of certain genes at the mRNA level [104–106]. Taking a specific RNA binding gene as an example, the TET RNA-binding proteins, EWSR1 and TAF15, have both been confirmed to be essential for the initiation and progression of the leukemia cell line [104, 107, 108].

### 3.4 Analysis of important KEGG pathways

Among the 345 features, three of them were about KEGG pathways. They were hsa03020 (RNA polymerase), hsa03015 (mRNA surveillance pathway) and hsa03013 (RNA transport). All three KEGG pathways turned out to involve in RNA metabolism. Among them, **hsa03020**, which describes the RNA polymerase associated pathway, has been screened out to be one of the essential pathways. As is known, RNA polymerase turns out to be the key enzyme that produces primary transcript RNAs in all organisms and many viruses [109]. Considering that gene transcription and expression turn out to be the essential procedures for the production of proteins and are major participants of most biological processes, it is reasonable to regard such KEGG pathways as a specific essential biological process for leukemia cells. The other two KEGG pathways, **hsa03015** and **hsa03013**, have both been related to RNA metabolism, validating the specific role of RNA metabolism for cell survival, especially the survival of leukemia cells, which has been validated by recent publications [110–112]. Describing the mRNA surveillance pathway, hsa03015 turns out to be the quality control mechanism that detects and degrades abnormal mRNAs [113]. For the specific biological functions of such KEGG pathways in leukemia cells, it has been confirmed that Human-T-cell Leukemia Virus type-I (HTLV-1) interferes with the normal mRNA decay processes and initiates the malignant transformation of leukemia cells [114]. Considering that the malignant transformation is significant for the survival of leukemia cells, it is reasonable to regard hsa03015 as a specific essential pathway. For hsa03013, it describes another specific process for the transcription of mRNAs, the RNA transport. Referring to the RNA transport from the nucleus to the cytoplasm, such biological processes describe the previous process of hsa03020 in the complete transcription process in cells, validating such biological processes as essential as hsa03020, as which we have analyzed above [109]. In addition, hsa03013 received the highest ranks in the MaxRel feature list (see S3 Table) among all KEGG pathways, suggesting it may be the most important pathway for classification of essential and non-essential genes.

In summary, these three KEGG pathways all describe RNA metabolism associated biological processes that have been confirmed to be significant for the malignant transformation and cell survival of leukemia cells, according to recent publications.

### 3.5 Further validation of the proposed prediction model

In this study, we used the enrichment scores of GO terms and KEGG pathways to represent each essential and non-essential gene. However, it can be seen that features of KEGG pathways were much less than those of GO terms. And in the optimal prediction model, only three features of KEGG pathways were involved. To test the contribution of KEGG pathway features, each essential and non-essential gene was represented by 279 features of KEGG pathways calculated by Eq 2. Then, the SVM algorithm was executed on these genes with its performance evaluated by ten-fold cross-validation. The predicted results were counted as SN, SP, ACC and MCC, listed in Table 1. It can be seen that these values are 0.873, 0.989, 0.966 and 0.891, respectively. They are not better than those yielded by the optimal prediction model. However, it is still quite good.

One of the contributions of this study was to propose a prediction model for classification of essential and non-essential genes. To give more clues for biologists, this model was further adopted to test the unlabeled genes that had not been validated to be essential genes or non-essential genes. As a result, 2,576 genes were predicted to be essential genes, which are provided in S6 Table. It is hopeful that many of them can be validated by solid evidences.

## 4. Conclusions

Based on the recent screening of essential/non-essential genes in the leukemia cell line K562, we continued the investigation using GO terms and KEGG pathways. Some important GO terms and KEGG pathways were extracted through a number of computational methods, such as the mRMR method, IFS method and SVM algorithm. In addition, a prediction model with nearly perfect performance was built. Some of the most important GO terms were extensively analyzed, and the results partly explain why they are essential for distinguishing essential and non-essential genes. It is hopeful that the identified GO terms and KEGG pathways may give new insights for further study of essential genes. Finally, we hope that our machine learning method can be applied to address other related problems, such as DNA-binding protein prediction [115], detection of tubule boundary [116], methylation site prediction [117], phosphorylation site prediction [118], and protein-protein interaction prediction [119].

## Supporting information

S1 Table Essential and non-essential genes. (DOCX)

S2 Table The mRMR feature list yielded by the mRMR method. (XLSX)

S3 Table The MaxRel feature list yielded by the mRMR method. (XLSX)

S4 Table The measurements yielded by the IFS method on different feature sets. (DOCX)

S5 Table Detailed information of the corresponding GO terms of the top 21 features in the mRMR feature list. (DOCX)

S6 Table The unlabeled genes that were predicted to be essential genes by the optimal prediction model. (DOCX)
