# Supplementary material for: Prediction and analysis of essential genes using the enrichments of gene ontology and KEGG pathways
Source: PLoS One. 2017 Sep 5;12(9):e0184129. doi: 10.1371/journal.pone.0184129 (PMC5584762; doi:10.1371/journal.pone.0184129)
Supplement: S1 Table — (DOCX) [file pone.0184129.s001.docx]

**S1 Table.** Essential and non-essential genes

| **Gene name** | **Tag** ^a^ |
| --- | --- |
| ALYREF | + |
| AQR | + |
| ARCN1 | + |
| BRIX1 | + |
| C12orf66 | + |
| CCT3 | + |
| CCT4 | + |
| CCT6A | + |
| CCT7 | + |
| CCT8 | + |
| CDC40 | + |
| CDC5L | + |
| CDK17 | + |
| CHD4 | + |
| CHMP2A | + |
| CLTC | + |
| COPA | + |
| COPB1 | + |
| COPS2 | + |
| COPS4 | + |
| COPS6 | + |
| COPS8 | + |
| COPZ1 | + |
| CSE1L | + |
| CTDNEP1 | + |
| DDB1 | + |
| DDX18 | + |
| DDX46 | + |
| DDX49 | + |
| DDX51 | + |
| DNM2 | + |
| DYNC1H1 | + |
| DYNC1I2 | + |
| EEF2 | + |
| EFTUD2 | + |
| EIF1AX | + |
| EIF2B4 | + |
| EIF2S2 | + |
| EIF3A | + |
| EIF3B | + |
| EIF3C | + |
| EIF3D | + |
| EIF3F | + |
| EIF3G | + |
| EIF3I | + |
| EIF4A3 | + |
| EIF5B | + |
| ERH | + |
| ETF1 | + |
| EXOSC10 | + |
| FTSJ3 | + |
| GAR1 | + |
| GNL3 | + |
| GTF3C4 | + |
| HAUS7 | + |
| HEATR1 | + |
| HNRNPC | + |
| HNRNPK | + |
| HNRNPM | + |
| HNRNPU | + |
| HSPA9 | + |
| HSPE1 | + |
| INTS9 | + |
| KARS | + |
| KPNB1 | + |
| LIAS | + |
| LSM4 | + |
| LSM6 | + |
| MRPS31 | + |
| MSANTD3 | + |
| NACA | + |
| NAPA | + |
| NAPG | + |
| NEDD8 | + |
| NHP2L1 | + |
| NUDT21 | + |
| NUP133 | + |
| NUP205 | + |
| NUP54 | + |
| NUP93 | + |
| NUP98 | + |
| NXF1 | + |
| PABPN1 | + |
| PAPOLA | + |
| PFDN2 | + |
| PHB | + |
| PHB2 | + |
| PHF5A | + |
| POLR2D | + |
| POLR2F | + |
| POLR2I | + |
| PPP2R1A | + |
| PRKAB2 | + |
| PRPF18 | + |
| PRPF19 | + |
| PRPF3 | + |
| PRPF31 | + |
| PRPF38A | + |
| PRPF8 | + |
| PRUNE | + |
| PSMA1 | + |
| PSMA2 | + |
| PSMA3 | + |
| PSMA6 | + |
| PSMB2 | + |
| PSMB3 | + |
| PSMC1 | + |
| PSMC2 | + |
| PSMC4 | + |
| PSMD1 | + |
| PSMD11 | + |
| PSMD7 | + |
| QARS | + |
| RAN | + |
| RBM17 | + |
| RILPL2 | + |
| RPA1 | + |
| RPA2 | + |
| RPL10 | + |
| RPL10A | + |
| RPL11 | + |
| RPL12 | + |
| RPL13 | + |
| RPL13A | + |
| RPL14 | + |
| RPL18 | + |
| RPL18A | + |
| RPL19 | + |
| RPL23A | + |
| RPL24 | + |
| RPL26 | + |
| RPL27 | + |
| RPL3 | + |
| RPL30 | + |
| RPL31 | + |
| RPL32 | + |
| RPL34 | + |
| RPL35 | + |
| RPL35A | + |
| RPL36 | + |
| RPL37 | + |
| RPL37A | + |
| RPL38 | + |
| RPL4 | + |
| RPL5 | + |
| RPL6 | + |
| RPL7 | + |
| RPL7A | + |
| RPLP0 | + |
| RPLP2 | + |
| RPN2 | + |
| RPS11 | + |
| RPS13 | + |
| RPS14 | + |
| RPS15 | + |
| RPS15A | + |
| RPS17 | + |
| RPS18 | + |
| RPS19 | + |
| RPS20 | + |
| RPS24 | + |
| RPS26 | + |
| RPS27A | + |
| RPS3 | + |
| RPS3A | + |
| RPS4X | + |
| RPS5 | + |
| RPS6 | + |
| RPS7 | + |
| RPS8 | + |
| RPS9 | + |
| RPSA | + |
| RRM1 | + |
| RUVBL1 | + |
| RUVBL2 | + |
| SDAD1 | + |
| SF3A1 | + |
| SF3A2 | + |
| SF3B1 | + |
| SF3B2 | + |
| SF3B3 | + |
| SF3B4 | + |
| SF3B5 | + |
| SFPQ | + |
| SHFM1 | + |
| SMC3 | + |
| SNRNP200 | + |
| SNRNP27 | + |
| SNRPD1 | + |
| SNRPD2 | + |
| SRCAP | + |
| SRFBP1 | + |
| SRSF1 | + |
| SRSF3 | + |
| SUPT5H | + |
| SUPT6H | + |
| SUPV3L1 | + |
| TFIP11 | + |
| THOP1 | + |
| TIMM10 | + |
| TSTA3 | + |
| TUBA1B | + |
| TUBGCP2 | + |
| U2AF1 | + |
| U2AF2 | + |
| UBA1 | + |
| USP39 | + |
| VCP | + |
| WDR12 | + |
| WDR60 | + |
| WDR61 | + |
| XAB2 | + |
| XPO1 | + |
| YY1 | + |
| ZBTB48 | + |
| ZC3H13 | + |
| ZNF207 | + |
| ABCG8 | - |
| ACCSL | - |
| ACTL7A | - |
| ACTL7B | - |
| ACTL9 | - |
| ACTRT1 | - |
| ADAD1 | - |
| ADAM18 | - |
| ADAM2 | - |
| ADAM20 | - |
| ADAM30 | - |
| ADH7 | - |
| AFM | - |
| AICDA | - |
| AIPL1 | - |
| ALPI | - |
| ALPPL2 | - |
| ALX3 | - |
| AMELX | - |
| ANKRD30A | - |
| ANKRD60 | - |
| ANTXRL | - |
| APOA4 | - |
| APOBEC1 | - |
| APOF | - |
| AQP12A | - |
| AQP8 | - |
| ARGFX | - |
| ART1 | - |
| ASB17 | - |
| ASIC5 | - |
| ASZ1 | - |
| ATOH1 | - |
| ATP4B | - |
| ATP6V1G3 | - |
| AWAT1 | - |
| AWAT2 | - |
| B3GNT6 | - |
| BANF2 | - |
| BARHL1 | - |
| BEND2 | - |
| BHLHE23 | - |
| BIRC8 | - |
| BMP10 | - |
| BMP15 | - |
| BPIFA1 | - |
| BPIFA3 | - |
| BPIFB3 | - |
| BPIFB6 | - |
| BPIFC | - |
| BPY2 | - |
| BRDT | - |
| BSND | - |
| C10orf113 | - |
| C10orf120 | - |
| C10orf53 | - |
| C11orf40 | - |
| C12orf40 | - |
| C14orf183 | - |
| C15orf55 | - |
| C16orf78 | - |
| C17orf102 | - |
| C17orf78 | - |
| C18orf26 | - |
| C19orf45 | - |
| C1orf146 | - |
| C20orf173 | - |
| C20orf203 | - |
| C20orf79 | - |
| C2orf57 | - |
| C2orf61 | - |
| C2orf71 | - |
| C2orf83 | - |
| C3orf30 | - |
| C4orf40 | - |
| C5orf20 | - |
| C6orf10 | - |
| C7orf66 | - |
| C7orf71 | - |
| C8A | - |
| C8B | - |
| C8orf17 | - |
| C8orf86 | - |
| C9orf53 | - |
| CABP2 | - |
| CABP5 | - |
| CABS1 | - |
| CACNG2 | - |
| CACNG3 | - |
| CACNG5 | - |
| CATSPER4 | - |
| CCDC155 | - |
| CCDC172 | - |
| CCDC83 | - |
| CCKAR | - |
| CCL1 | - |
| CCT8L2 | - |
| CD200R1L | - |
| CDCP2 | - |
| CDX2 | - |
| CDX4 | - |
| CDY1 | - |
| CDY1B | - |
| CDY2A | - |
| CDY2B | - |
| CEACAM7 | - |
| CELA2A | - |
| CELA3A | - |
| CELA3B | - |
| CER1 | - |
| CETN1 | - |
| CFHR2 | - |
| CFHR5 | - |
| CHAT | - |
| CHRNA6 | - |
| CHRNB3 | - |
| CLCA1 | - |
| CLDN17 | - |
| CLEC2A | - |
| CLEC3A | - |
| CLEC6A | - |
| CLRN1 | - |
| CNBD1 | - |
| CNGA2 | - |
| CNGB3 | - |
| CNPY1 | - |
| CNTNAP5 | - |
| COL20A1 | - |
| COX7B2 | - |
| CPXCR1 | - |
| CRNN | - |
| CRX | - |
| CRYGB | - |
| CSH1 | - |
| CSHL1 | - |
| CSN2 | - |
| CSN3 | - |
| CST11 | - |
| CST4 | - |
| CST5 | - |
| CST8 | - |
| CST9 | - |
| CST9L | - |
| CSTL1 | - |
| CT45A2 | - |
| CT45A4 | - |
| CT45A5 | - |
| CT47A11 | - |
| CTCFL | - |
| CTRB1 | - |
| CXorf1 | - |
| CXorf66 | - |
| CYLC2 | - |
| CYP11B1 | - |
| CYP11B2 | - |
| CYP26C1 | - |
| CYP2A13 | - |
| CYP2C19 | - |
| CYP4A22 | - |
| CYP4F8 | - |
| CYP7A1 | - |
| DAZ1 | - |
| DAZ2 | - |
| DAZ3 | - |
| DAZ4 | - |
| DAZL | - |
| DCAF4L2 | - |
| DCAF8L1 | - |
| DDI1 | - |
| DDX4 | - |
| DEFA5 | - |
| DEFA6 | - |
| DEFB103B | - |
| DEFB104A | - |
| DEFB106A | - |
| DEFB107A | - |
| DEFB118 | - |
| DEFB123 | - |
| DEFB126 | - |
| DEFB127 | - |
| DEFB129 | - |
| DGAT2L6 | - |
| DGKK | - |
| DIRC1 | - |
| DMP1 | - |
| DMRT1 | - |
| DMRTB1 | - |
| DMRTC2 | - |
| DPCR1 | - |
| DPRX | - |
| DRD3 | - |
| DRGX | - |
| DSCR4 | - |
| DSG4 | - |
| DSPP | - |
| DTX2 | - |
| DUSP21 | - |
| DUX4 | - |
| DUX4L7 | - |
| DUXA | - |
| EFCAB3 | - |
| EGR4 | - |
| ENTHD1 | - |
| ESX1 | - |
| EVX1 | - |
| F13B | - |
| F9 | - |
| FABP2 | - |
| FAM106A | - |
| FAM47A | - |
| FAM47B | - |
| FAM47C | - |
| FAM71A | - |
| FAM71B | - |
| FAM71C | - |
| FAM75A7 | - |
| FAM75D1 | - |
| FCRL4 | - |
| FEZF1 | - |
| FEZF2 | - |
| FFAR1 | - |
| FGF3 | - |
| FGF4 | - |
| FGF6 | - |
| FIGLA | - |
| FLG2 | - |
| FMR1NB | - |
| FNDC7 | - |
| FNDC9 | - |
| FOXB1 | - |
| FOXB2 | - |
| FOXD4L3 | - |
| FOXD4L4 | - |
| FOXE3 | - |
| FOXN1 | - |
| FOXR1 | - |
| FRG2 | - |
| FRMD7 | - |
| FSCB | - |
| FUT5 | - |
| FUT9 | - |
| G6PC | - |
| GABRA1 | - |
| GABRA6 | - |
| GAGE1 | - |
| GAGE2C | - |
| GALNTL5 | - |
| GALR1 | - |
| GALR3 | - |
| GBP7 | - |
| GCG | - |
| GCM2 | - |
| GDF2 | - |
| GFRA4 | - |
| GFRAL | - |
| GH2 | - |
| GHRH | - |
| GHSR | - |
| GIF | - |
| GJA10 | - |
| GJA8 | - |
| GK2 | - |
| GKN2 | - |
| GLRA1 | - |
| GLRA2 | - |
| GLT6D1 | - |
| GML | - |
| GOLGA6L2 | - |
| GOT1L1 | - |
| GPR101 | - |
| GPR111 | - |
| GPR119 | - |
| GPR128 | - |
| GPR139 | - |
| GPR144 | - |
| GPR148 | - |
| GPR151 | - |
| GPR152 | - |
| GPR26 | - |
| GPR31 | - |
| GPR32 | - |
| GPR45 | - |
| GPR50 | - |
| GPR52 | - |
| GPR78 | - |
| GPRC6A | - |
| GPX5 | - |
| GPX6 | - |
| GRK1 | - |
| GRM4 | - |
| GRM5 | - |
| GRM6 | - |
| GSC2 | - |
| GSTA5 | - |
| GSX1 | - |
| GSX2 | - |
| GUCA2A | - |
| GUCY2F | - |
| H1FOO | - |
| H2BFM | - |
| H2BFWT | - |
| HAO1 | - |
| HCRTR2 | - |
| HDGFL1 | - |
| HHLA1 | - |
| HIST1H2AA | - |
| HIST1H2BA | - |
| HIST1H4G | - |
| HMX1 | - |
| HOXB1 | - |
| HOXD12 | - |
| HRG | - |
| HRH3 | - |
| HSFY1 | - |
| HSFY2 | - |
| HTN3 | - |
| HTR1A | - |
| HTR2C | - |
| HTR3C | - |
| HTR3D | - |
| HTR3E | - |
| HTR5A | - |
| HTR6 | - |
| IAPP | - |
| IFIT1B | - |
| IFNA10 | - |
| IFNA14 | - |
| IFNA16 | - |
| IFNA17 | - |
| IFNA2 | - |
| IFNA21 | - |
| IFNA4 | - |
| IFNA5 | - |
| IFNA6 | - |
| IFNA7 | - |
| IFNA8 | - |
| IFNB1 | - |
| IFNK | - |
| IFNW1 | - |
| IL12B | - |
| IL13 | - |
| IL17A | - |
| IL17F | - |
| IL1F10 | - |
| IL21 | - |
| IL22 | - |
| IL25 | - |
| IL26 | - |
| IL28A | - |
| IL28B | - |
| IL29 | - |
| IL3 | - |
| IL31 | - |
| IL36A | - |
| IL36B | - |
| IL36RN | - |
| IL9 | - |
| INS | - |
| INSL5 | - |
| INSL6 | - |
| INSM2 | - |
| INSRR | - |
| IQCF1 | - |
| IRGC | - |
| ISX | - |
| ITIH6 | - |
| IZUMO2 | - |
| KCNA10 | - |
| KCNB2 | - |
| KCNG4 | - |
| KCNK10 | - |
| KCNK16 | - |
| KCNK18 | - |
| KCNV1 | - |
| KHDC3L | - |
| KIF2B | - |
| KIR2DL1 | - |
| KIR3DL3 | - |
| KLK12 | - |
| KLK9 | - |
| KRT2 | - |
| KRT25 | - |
| KRT26 | - |
| KRT28 | - |
| KRT33A | - |
| KRT35 | - |
| KRT36 | - |
| KRT37 | - |
| KRT38 | - |
| KRT40 | - |
| KRT71 | - |
| KRT73 | - |
| KRT74 | - |
| KRT75 | - |
| KRT76 | - |
| KRT77 | - |
| KRT78 | - |
| KRT82 | - |
| KRT84 | - |
| KRT85 | - |
| KRT86 | - |
| KRT9 | - |
| KRTAP1-1 | - |
| KRTAP10-1 | - |
| KRTAP10-10 | - |
| KRTAP10-11 | - |
| KRTAP10-12 | - |
| KRTAP10-2 | - |
| KRTAP10-4 | - |
| KRTAP10-5 | - |
| KRTAP10-6 | - |
| KRTAP10-7 | - |
| KRTAP10-8 | - |
| KRTAP10-9 | - |
| KRTAP11-1 | - |
| KRTAP13-1 | - |
| KRTAP13-2 | - |
| KRTAP13-3 | - |
| KRTAP13-4 | - |
| KRTAP15-1 | - |
| KRTAP17-1 | - |
| KRTAP19-3 | - |
| KRTAP23-1 | - |
| KRTAP26-1 | - |
| KRTAP3-2 | - |
| KRTAP4-11 | - |
| KRTAP4-12 | - |
| KRTAP4-2 | - |
| KRTAP4-4 | - |
| KRTAP4-7 | - |
| KRTAP5-2 | - |
| KRTAP9-2 | - |
| KRTAP9-3 | - |
| KRTAP9-4 | - |
| LALBA | - |
| LBX1 | - |
| LCN9 | - |
| LCT | - |
| LGALS13 | - |
| LGALS14 | - |
| LHFPL5 | - |
| LHX3 | - |
| LHX5 | - |
| LIM2 | - |
| LIN28A | - |
| LIPM | - |
| LOR | - |
| LRIT1 | - |
| LRIT2 | - |
| LRRC10 | - |
| LUZP4 | - |
| LYZL1 | - |
| LYZL2 | - |
| LYZL6 | - |
| MAGEA10 | - |
| MAGEA11 | - |
| MAGEB1 | - |
| MAGEB10 | - |
| MAGEB18 | - |
| MAGEB3 | - |
| MAGEB4 | - |
| MAGEC3 | - |
| MAS1 | - |
| MAS1L | - |
| MBD3L1 | - |
| MBD3L2 | - |
| MBL2 | - |
| MC2R | - |
| MC3R | - |
| MC5R | - |
| MEP1A | - |
| MEP1B | - |
| MEPE | - |
| MFRP | - |
| MMD2 | - |
| MMP20 | - |
| MMP21 | - |
| MMP26 | - |
| MMP27 | - |
| MOGAT3 | - |
| MORC1 | - |
| MRGPRD | - |
| MRGPRX1 | - |
| MRGPRX2 | - |
| MRGPRX4 | - |
| MS4A10 | - |
| MS4A13 | - |
| MS4A5 | - |
| MSGN1 | - |
| MT1B | - |
| MTNR1B | - |
| MUC17 | - |
| MUC7 | - |
| MYBPC3 | - |
| MYF5 | - |
| NANOGNB | - |
| NANOS2 | - |
| NCR2 | - |
| NDST4 | - |
| NEUROD2 | - |
| NEUROD4 | - |
| NEUROD6 | - |
| NEUROG1 | - |
| NKX2-1 | - |
| NKX2-2 | - |
| NLRP4 | - |
| NLRP5 | - |
| NLRP8 | - |
| NLRP9 | - |
| NMS | - |
| NOBOX | - |
| NOTO | - |
| NOX3 | - |
| NPFFR1 | - |
| NPHS2 | - |
| NPSR1 | - |
| NPVF | - |
| NR2E1 | - |
| NYX | - |
| OC90 | - |
| OLIG2 | - |
| OLIG3 | - |
| OPALIN | - |
| OPN1LW | - |
| OPN5 | - |
| OR10A2 | - |
| OR10A4 | - |
| OR10A5 | - |
| OR10H1 | - |
| OR10H2 | - |
| OR10H3 | - |
| OR10J1 | - |
| OR10R2 | - |
| OR10S1 | - |
| OR10X1 | - |
| OR10Z1 | - |
| OR11A1 | - |
| OR12D2 | - |
| OR12D3 | - |
| OR13C3 | - |
| OR13D1 | - |
| OR14A16 | - |
| OR1A1 | - |
| OR1A2 | - |
| OR1B1 | - |
| OR1D2 | - |
| OR1E1 | - |
| OR1E2 | - |
| OR1G1 | - |
| OR1L6 | - |
| OR1N2 | - |
| OR1S1 | - |
| OR1S2 | - |
| OR2AK2 | - |
| OR2AT4 | - |
| OR2C1 | - |
| OR2C3 | - |
| OR2D2 | - |
| OR2D3 | - |
| OR2F1 | - |
| OR2G2 | - |
| OR2G3 | - |
| OR2H1 | - |
| OR2J2 | - |
| OR2L3 | - |
| OR2T1 | - |
| OR2T10 | - |
| OR2T12 | - |
| OR2T2 | - |
| OR2T27 | - |
| OR2T33 | - |
| OR2T4 | - |
| OR2T5 | - |
| OR2W1 | - |
| OR3A1 | - |
| OR3A2 | - |
| OR3A3 | - |
| OR4C11 | - |
| OR4C3 | - |
| OR4D1 | - |
| OR4D10 | - |
| OR4D11 | - |
| OR4D9 | - |
| OR4K17 | - |
| OR51B6 | - |
| OR51D1 | - |
| OR51F2 | - |
| OR51T1 | - |
| OR51V1 | - |
| OR52A1 | - |
| OR52A5 | - |
| OR52B2 | - |
| OR52B6 | - |
| OR52E8 | - |
| OR52I2 | - |
| OR52K2 | - |
| OR52L1 | - |
| OR52M1 | - |
| OR52R1 | - |
| OR52W1 | - |
| OR56A1 | - |
| OR56A4 | - |
| OR56B1 | - |
| OR5AU1 | - |
| OR5C1 | - |
| OR5I1 | - |
| OR5M1 | - |
| OR5M10 | - |
| OR5P2 | - |
| OR5P3 | - |
| OR5R1 | - |
| OR5T1 | - |
| OR5T2 | - |
| OR5T3 | - |
| OR5V1 | - |
| OR5W2 | - |
| OR6A2 | - |
| OR6K6 | - |
| OR6S1 | - |
| OR6V1 | - |
| OR7A17 | - |
| OR7C2 | - |
| OR7D4 | - |
| OR7G2 | - |
| OR8A1 | - |
| OR8B8 | - |
| OR8G5 | - |
| OR8U1 | - |
| OR9Q2 | - |
| OTOP1 | - |
| OTOP3 | - |
| OTOR | - |
| OTP | - |
| OTUD6A | - |
| OTX2 | - |
| PAGE3 | - |
| PANX3 | - |
| PASD1 | - |
| PAX1 | - |
| PAX4 | - |
| PBOV1 | - |
| PDCL2 | - |
| PDE6H | - |
| PDILT | - |
| PDX1 | - |
| PDYN | - |
| PGK2 | - |
| PGLYRP2 | - |
| PGLYRP3 | - |
| PIWIL1 | - |
| PIWIL3 | - |
| PKD1L3 | - |
| PLA2G2E | - |
| PLA2G2F | - |
| PLA2G4E | - |
| PLAC1L | - |
| PNLIP | - |
| PNLIPRP1 | - |
| PNLIPRP2 | - |
| PNPLA5 | - |
| POM121L12 | - |
| POTEA | - |
| POTED | - |
| POTEG | - |
| POTEH | - |
| POU3F4 | - |
| POU4F2 | - |
| POU4F3 | - |
| POU5F2 | - |
| PPP3R2 | - |
| PRAMEF1 | - |
| PRAMEF19 | - |
| PRAMEF2 | - |
| PRAMEF3 | - |
| PRAMEF4 | - |
| PRAMEF7 | - |
| PRB1 | - |
| PRB4 | - |
| PRDM13 | - |
| PRDM14 | - |
| PRDM7 | - |
| PRDM9 | - |
| PRG3 | - |
| PRLH | - |
| PRLHR | - |
| PROP1 | - |
| PRSS33 | - |
| PRSS37 | - |
| PRSS38 | - |
| PRSS41 | - |
| PRSS55 | - |
| PRSS58 | - |
| PRY2 | - |
| PSKH2 | - |
| PTF1A | - |
| RAX | - |
| RAX2 | - |
| RBM46 | - |
| RBMXL2 | - |
| RBMY1A1 | - |
| RBMY1B | - |
| RBMY1D | - |
| RBMY1E | - |
| RBMY1F | - |
| RBMY1J | - |
| RBP3 | - |
| RBPJL | - |
| RD3 | - |
| RDH8 | - |
| REG3A | - |
| RESP18 | - |
| RETNLB | - |
| REXO1L1 | - |
| RFPL3 | - |
| RFPL4B | - |
| RFX6 | - |
| RHO | - |
| RHOXF2 | - |
| RNASE10 | - |
| RNASE11 | - |
| RNASE12 | - |
| RNASE13 | - |
| RNASE8 | - |
| RNASE9 | - |
| RND2 | - |
| RNF113B | - |
| RNF17 | - |
| RP1 | - |
| RP1L1 | - |
| RPE65 | - |
| RPTN | - |
| RS1 | - |
| RTP1 | - |
| RTP2 | - |
| RXFP2 | - |
| RXFP3 | - |
| S100A7A | - |
| S100G | - |
| SAGE1 | - |
| SAMD7 | - |
| SCGB1D1 | - |
| SCN10A | - |
| SCRT2 | - |
| SDR9C7 | - |
| SEC14L3 | - |
| SEMG2 | - |
| 14-Sep | - |
| SERPINA12 | - |
| SERPINA7 | - |
| SERPINA9 | - |
| SERPINB12 | - |
| SHCBP1L | - |
| SHOX | - |
| SI | - |
| SIGLECL1 | - |
| SIX6 | - |
| SLC10A2 | - |
| SLC13A1 | - |
| SLC17A2 | - |
| SLC17A6 | - |
| SLC18A3 | - |
| SLC22A12 | - |
| SLC22A13 | - |
| SLC22A24 | - |
| SLC22A25 | - |
| SLC22A6 | - |
| SLC22A8 | - |
| SLC22A9 | - |
| SLC25A2 | - |
| SLC25A31 | - |
| SLC2A2 | - |
| SLC2A7 | - |
| SLC32A1 | - |
| SLC34A1 | - |
| SLC36A3 | - |
| SLC39A12 | - |
| SLC6A18 | - |
| SLC6A5 | - |
| SLC6A7 | - |
| SLC7A13 | - |
| SLCO1B1 | - |
| SLCO6A1 | - |
| SLITRK1 | - |
| SOHLH1 | - |
| SOX1 | - |
| SOX14 | - |
| SP8 | - |
| SPACA1 | - |
| SPACA5 | - |
| SPACA7 | - |
| SPATA16 | - |
| SPATA21 | - |
| SPEM1 | - |
| SPHAR | - |
| SPINK14 | - |
| SPO11 | - |
| SPPL2C | - |
| SPRR4 | - |
| SSTR4 | - |
| SSX3 | - |
| SSX5 | - |
| SSX7 | - |
| SSX8 | - |
| SSX9 | - |
| STATH | - |
| SULT6B1 | - |
| SUN5 | - |
| T | - |
| TAAR1 | - |
| TAAR2 | - |
| TAAR5 | - |
| TAAR6 | - |
| TAAR8 | - |
| TAAR9 | - |
| TAS1R2 | - |
| TAS2R1 | - |
| TAS2R13 | - |
| TAS2R16 | - |
| TAS2R39 | - |
| TAS2R40 | - |
| TAS2R41 | - |
| TAS2R42 | - |
| TAS2R43 | - |
| TAS2R46 | - |
| TAS2R50 | - |
| TAS2R60 | - |
| TAS2R7 | - |
| TAS2R8 | - |
| TAS2R9 | - |
| TBC1D21 | - |
| TBC1D29 | - |
| TBL1Y | - |
| TBPL2 | - |
| TBR1 | - |
| TBX10 | - |
| TCEB3B | - |
| TCEB3C | - |
| TCHHL1 | - |
| TCP10L2 | - |
| TEDDM1 | - |
| TEX101 | - |
| TEX13A | - |
| TEX28 | - |
| TEX34 | - |
| TFAP2D | - |
| TFDP3 | - |
| TGIF2LX | - |
| TGIF2LY | - |
| TGM6 | - |
| TKTL2 | - |
| TLX1 | - |
| TMEM132D | - |
| TMEM174 | - |
| TMEM207 | - |
| TMEM225 | - |
| TMIGD1 | - |
| TMPRSS11A | - |
| TMPRSS11B | - |
| TMPRSS11F | - |
| TMPRSS12 | - |
| TMPRSS15 | - |
| TNR | - |
| TPD52L3 | - |
| TPH2 | - |
| TPRX1 | - |
| TPTE | - |
| TREML4 | - |
| TRHR | - |
| TRIM40 | - |
| TRIM42 | - |
| TRIM43 | - |
| TRIM48 | - |
| TRIM49 | - |
| TRIM51 | - |
| TRIM60 | - |
| TRIM67 | - |
| TRIML1 | - |
| TRPC5 | - |
| TRPC7 | - |
| TRPM1 | - |
| TRPV5 | - |
| TSGA13 | - |
| TSHB | - |
| TSPAN16 | - |
| TSPO2 | - |
| TSPY1 | - |
| TSPYL6 | - |
| TSSK1B | - |
| TSSK2 | - |
| TXNDC8 | - |
| TYR | - |
| UBQLN3 | - |
| UMOD | - |
| UROC1 | - |
| USP17L2 | - |
| USP26 | - |
| USP29 | - |
| UTS2R | - |
| VAX1 | - |
| VCX3A | - |
| VHLL | - |
| VN1R2 | - |
| VN1R4 | - |
| VN1R5 | - |
| VPREB1 | - |
| VRTN | - |
| VSX2 | - |
| WFDC10A | - |
| WFDC11 | - |
| WFDC9 | - |
| XAGE2 | - |
| XAGE5 | - |
| XKR7 | - |
| ZAN | - |
| ZCCHC13 | - |
| ZCCHC16 | - |
| ZG16 | - |
| ZIC3 | - |
| ZIM3 | - |
| ZNF645 | - |
| ZNF648 | - |
| ZNF679 | - |
| ZNF804B | - |
| ZNRF4 | - |
| ZP2 | - |
| ZP4 | - |
| ZSWIM2 | - |

a: This column indicates whether the gene is an essential gene or a non-essential one, “+” represents essential gene and “-” represents non-essential gene.
