# Supplementary material for: Prediction and analysis of essential genes using the enrichments of gene ontology and KEGG pathways
Source: PLoS One. 2017 Sep 5;12(9):e0184129. doi: 10.1371/journal.pone.0184129 (PMC5584762; doi:10.1371/journal.pone.0184129)
Supplement: S5 Table — (DOCX) [file pone.0184129.s005.docx]

**S5 Table.** Detailed information of the corresponding GO terms of the top 21 features in the mRMR feature list

| **Rank in mRMR feature list** | **GO term ID** | **GO term** | **Cluster** | **Description** |
| --- | --- | --- | --- | --- |
| 1 | GO:0032991 | macromolecular complex | cellular component | A stable assembly of two or more macromolecules, i.e. proteins, nucleic acids, carbohydrates or lipids, in which the constituent parts function together. |
| 2 | GO:0021888 | hypothalamus gonadotrophin-releasing hormone neuron development | biological process | The process whose specific outcome is the progression of a hypothalamus gonadotrophin-releasing hormone neuron over time, from initial commitment of its fate, to the fully functional differentiated cell |
| 3 | GO:0071008 | U2-type post-mRNA release spliceosomal complex | cellular component | A spliceosomal complex that is formed following the release of the spliced product from the post-spliceosomal complex and contains the excised intron and the U2, U5 and U6 snRNPs. |
| 4 | GO:0044424 | intracellular part | cellular component | Any constituent part of the living contents of a cell; the matter contained within (but not including) the plasma membrane, usually taken to exclude large vacuoles and masses of secretory or ingested material. In eukaryotes it includes the nucleus and cytoplasm. |
| 5 | GO:0000154 | rRNA modification | biological process | The covalent alteration of one or more nucleotides within an rRNA molecule to produce an rRNA molecule with a sequence that differs from that coded genetically. |
| 6 | GO:0043226 | organelle | cellular component | Organized structure of distinctive morphology and function. Includes the nucleus, mitochondria, plastids, vacuoles, vesicles, ribosomes and the cytoskeleton, and prokaryotic structures such as anammoxosomes and pirellulosomes. Excludes the plasma membrane. |
| 7 | GO:0016071 | mRNA metabolic process | biological process | The chemical reactions and pathways involving mRNA, messenger RNA, which is responsible for carrying the coded genetic 'message', transcribed from DNA, to sites of protein assembly at the ribosomes. |
| 8 | GO:0071146 | SMAD3-SMAD4 protein complex | cellular component | A heteromeric SMAD protein complex that contains SMAD3 and SMAD4 |
| 9 | GO:0072669 | tRNA-splicing ligase complex | cellular component | A protein complex that catalyzes the ligation of cleaved pre-tRNAs by directly joining spliced tRNA halves to mature-sized tRNAs by incorporating the precursor-derived splice junction phosphate into the mature tRNA as a canonical 3',5'-phosphodiester. |
| 10 | GO:0044422 | organelle part | cellular component | Any constituent part of an organelle, an organized structure of distinctive morphology and function. Includes constituent parts of the nucleus, mitochondria, plastids, vacuoles, vesicles, ribosomes and the cytoskeleton, but excludes the plasma membrane. |
| 11 | GO:0021886 | hypothalamus gonadotrophin-releasing hormone neuron differentiation | biological process | The process in which a relatively unspecialized cell acquires specialized features of a neuron located in the hypothalamus. These neurons release gonadotrophin-releasing hormone as a neural transmitter |
| 12 | GO:0002183 | cytoplasmic translational initiation | biological process | The process preceding formation of the peptide bond between the first two amino acids of a protein in the cytoplasm. This includes the formation of a complex of the ribosome, mRNA, and an initiation complex that contains the first aminoacyl-tRNA. |
| 13 | GO:0005622 | intracellular | cellular component | The living contents of a cell; the matter contained within (but not including) the plasma membrane, usually taken to exclude large vacuoles and masses of secretory or ingested material. In eukaryotes it includes the nucleus and cytoplasm. |
| 14 | GO:0015030 | Cajal body | cellular component | A class of nuclear body, first seen after silver staining by Ramon y Cajal in 1903, enriched in small nuclear ribonucleoproteins, and certain general RNA polymerase II transcription factors; ultrastructurally, they appear as a tangle of coiled, electron-dense threads roughly 0.5 micrometers in diameter; involved in aspects of snRNP biogenesis; the protein coilin serves as a marker for Cajal bodies. Some argue that Cajal bodies are the sites for preassembly of transcriptosomes, unitary particles involved in transcription and processing of RNA. |
| 15 | GO:0030874 | nucleolar chromatin | cellular component | The portion of nuclear chromatin associated with the nucleolus; includes the DNA encoding the ribosomal RNA. |
| 16 | GO:0044446 | intracellular organelle part | cellular component | A constituent part of an intracellular organelle, an organized structure of distinctive morphology and function, occurring within the cell. Includes constituent parts of the nucleus, mitochondria, plastids, vacuoles, vesicles, ribosomes and the cytoskeleton but excludes the plasma membrane. |
| 17 | GO:0010467 | gene expression | biological process | The process in which a gene's sequence is converted into a mature gene product or products (proteins or RNA). This includes the production of an RNA transcript as well as any processing to produce a mature RNA product or an mRNA (for protein-coding genes) and the translation of that mRNA into protein. Protein maturation is included when required to form an active form of a product from an inactive precursor form. |
| 18 | GO:0043227 | membrane-bounded organelle | cellular component | Organized structure of distinctive morphology and function, bounded by a single or double lipid bilayer membrane. Includes the nucleus, mitochondria, plastids, vacuoles, and vesicles. Excludes the plasma membrane. |
| 19 | GO:1902369 | negative regulation of RNA catabolic process | biological process | Any process that stops, prevents or reduces the frequency, rate or extent of RNA catabolic process |
| 20 | GO:0044822 | poly(A) RNA binding | molecular function | Interacting non-covalently with a poly (A) RNA, a RNA molecule which has a tail of adenine bases. |
| 21 | GO:0005737 | cytoplasm | cellular component | All of the contents of a cell excluding the plasma membrane and nucleus, but including other subcellular structures. |
