# Supplementary material for: Prediction and analysis of essential genes using the enrichments of gene ontology and KEGG pathways
Source: PLoS One. 2017 Sep 5;12(9):e0184129. doi: 10.1371/journal.pone.0184129 (PMC5584762; doi:10.1371/journal.pone.0184129)
Supplement: S6 Table — (DOCX) [file pone.0184129.s006.docx]

**S6 Table.** The unlabeled genes that were predicted to be essential genes by the optimal prediction model.

| GORASP2 | C2orf49 | RAC2 | VAMP3 | SEPT2 |
| --- | --- | --- | --- | --- |
| PCNA | CCNG1 | STX12 | MAP1LC3B | SEPT7 |
| HIST2H4B | MARS2 | MRPL1 | SRSF7 | AAAS |
| DOCK4 | NCL | HNRNPH2 | RDH14 | ACBD6 |
| PPP6C | PGK1 | RARS | NELFB | ACTN4 |
| ELAVL1 | HIST1H4F | HSP90AA1 | TARDBP | ACTR10 |
| PNISR | NFU1 | UBE2L3 | HNRNPLL | ACTR6 |
| NKRF | SPTBN2 | PRKAA2 | CCDC73 | ACTR8 |
| MT-ND4L | SLC35B1 | TXN2 | PSMG2 | ADH5 |
| AK3 | NAA25 | KPNA4 | UBR5 | AGO1 |
| PIF1 | THOC2 | RRP1 | NUP62 | AKT1 |
| CSNK2B | SLTM | ACTR2 | NDUFS6 | ALG12 |
| KIAA0020 | HSPBP1 | COX17 | TPP2 | ALG8 |
| NUP35 | CBX1 | HINT2 | GEMIN6 | AP1M1 |
| HDAC8 | PA2G4 | SARNP | MRPL15 | AP3B1 |
| ANP32E | EPN2 | XRN2 | RAD23A | AP4B1 |
| UPF1 | MDM2 | DCTN1 | SAMM50 | AP5M1 |
| SEC23IP | USO1 | TNPO1 | ANKHD1 | APOA1BP |
| HNRNPR | AP3S1 | NNT | HDAC4 | ARF3 |
| VPS45 | MAPK3 | DCTN2 | TUBA3E | ARF4 |
| INTS1 | TRIM24 | PDIA3 | RPE | ARHGEF2 |
| AGO2 | RNF7 | EPRS | GTPBP4 | ARL6IP1 |
| ETHE1 | REXO2 | METAP2 | PAK1 | ARL8A |
| BZW1 | HAUS2 | DKC1 | TMEM147 | ASH2L |
| DNAJC5 | RBM7 | RABGGTB | SEC61B | ASPM |
| AP2S1 | ZW10 | CEBPZ | DDX41 | ASUN |
| MND1 | CEP55 | UFSP2 | PPP3CB | ATAD2 |
| CDKN1A | NDUFB6 | GGA1 | RNGTT | ATP5F1 |
| PSIP1 | ATR | CPSF1 | THUMPD2 | ATP5J2 |
| CSTF1 | PPM1G | ABCF2 | MCM3AP | ATP6V1F |
| CCNB1IP1 | RNF138 | KNSTRN | RAB2A | ATP6V1G1 |
| STXBP2 | CDKN2A | EIF2S1 | NDUFA5 | ATXN7L2 |
| EXOC6 | LARS | EXOC7 | NMNAT1 | BAZ1B |
| CTSD | DUSP11 | SUMO3 | NCOA6 | BCAP31 |
| PNPLA8 | DNAH8 | STX5 | ETFA | BCAS2 |
| SLC2A1 | FDXACB1 | GTF3C5 | GNG5 | BET1 |
| CCT6B | DBR1 | LDHA | LYAR | BET1L |
| MRPS21 | VPS72 | STRA13 | DHX30 | BIRC2 |
| VPS33B | MCM7 | CDK11A | DDX47 | BIRC5 |
| FAU | DDX19B | NOP14 | ROMO1 | BMI1 |
| PRRC2A | NCOA3 | SNRPG | MATR3 | BRCA1 |
| MED1 | NSMCE4A | NVL | LAMP1 | BRF1 |
| TMEM241 | HIST1H4J | USP14 | CSTF3 | BRF2 |
| NAA10 | ANAPC5 | SNRNP70 | NXF3 | BTBD1 |
| EWSR1 | HNRNPA0 | RRAS | UTP23 | BUD31 |
| DDX10 | NCKAP1 | EDF1 | MED30 | C10orf2 |
| MAD1L1 | SNRPA | RPL29 | SPCS1 | C11orf73 |
| CHD2 | NPLOC4 | NUP37 | MED10 | C12orf10 |
| EZR | NDC1 | PAIP1 | COX4I1 | C14orf142 |
| PARP4 | CDC45 | THOC1 | RBM34 | C14orf166 |
| DEK | WDR75 | NDUFS1 | KIAA0947 | C16orf80 |
| HADH | G3BP2 | MARCH7 | TFB2M | C19orf10 |
| RMND1 | NOC3L | NXT1 | GRPEL1 | C19orf70 |
| MRPL23 | NUFIP2 | LIN7C | DDX39A | C1orf112 |
| URB1 | UBE2I | INCENP | COA6 | C20orf24 |
| MRPS36 | YIPF5 | TIMM21 | RPL10L | C2CD5 |
| SEC24B | GHITM | SLK | MALSU1 | CACYBP |
| C1D | AURKB | TLN1 | MED16 | CANX |
| ANAPC10 | MRPS9 | FAM98A | XRCC6 | CBX3 |
| SAE1 | CD164 | CUL9 | MED12 | CCDC101 |
| POLR1B | RPSAP58 | UBE2E1 | DPH5 | CCDC43 |
| POLR3K | ANXA11 | CAPZA2 | TBCE | CCDC90B |
| SSR3 | PPP4C | CUL7 | HIST4H4 | CCDC94 |
| ARL5A | TOMM40 | NPM1 | NOL8 | CCNT1 |
| GMPS | HIGD1A | RNPS1 | HAX1 | CCNT2 |
| CUL3 | SPTAN1 | PGAM5 | MAPRE1 | CDC26 |
| SUGP2 | UQCRFS1 | MIS18BP1 | ZMYM3 | CDC73 |
| ZMPSTE24 | TMEM14B | RBM10 | ZNHIT6 | CDCA8 |
| KIAA1279 | EEF1D | ALG6 | PSMB5 | CDKN3 |
| IDH3B | COX7B | MRPL17 | TAF12 | CDT1 |
| EEF1E1 | PSMB1 | SHQ1 | NDUFS7 | CENPE |
| ASCC3 | HIST1H4D | CALM2 | ZNHIT1 | CENPK |
| ERAL1 | XPO7 | PRMT3 | DR1 | CEP57 |
| POLR3A | ARNT | SEC24D | PMVK | CETN2 |
| BOLA1 | SUGP1 | YARS2 | CENPN | CHCHD2 |
| KIF11 | PRKAA1 | MAD2L1 | CALR | CHCHD3 |
| STRBP | C12orf29 | MLST8 | NOL10 | CHEK1 |
| PLK4 | ATP5C1 | SKI | POLR2E | CHERP |
| ZC3H15 | ORMDL1 | PDCD2L | CKS2 | CHORDC1 |
| HMGB1 | NUP153 | ACTL6B | MBD3 | CHUK |
| POLR1C | PIK3R4 | GNL3L | PPM1D | CIB1 |
| HSPA13 | NAA15 | ERCC2 | ODC1 | CLASP2 |
| MRPL39 | NUP210 | EXOSC7 | PRKCSH | CLNS1A |
| NUDT1 | DNAJB11 | LETM1 | IMP3 | CLP1 |
| PSMD6 | NOB1 | SEC61A2 | ABCD3 | CLPP |
| YIF1A | GRWD1 | EXOSC9 | SPCS2 | CLUH |
| NMNAT3 | GATAD2A | SLBP | TWF1 | CMAS |
| STXBP1 | TAF1L | MELK | SERP1 | CNIH1 |
| PNO1 | KTI12 | CLTA | TMED2 | CNOT1 |
| METTL2A | HIST1H1A | TUT1 | RPF1 | CNOT3 |
| FIBP | HGS | ATP5A1 | COA4 | CNOT6 |
| HAUS6 | PPP2R2B | RABEP1 | STX17 | COMMD3 |
| DDX52 | CD2BP2 | TRAP1 | MAP3K7 | COPG1 |
| USP38 | RPP30 | UXT | TBRG4 | COPG2 |
| RANBP6 | AHSA1 | MAP1B | C12orf44 | COPS3 |
| NBN | YWHAH | PABPC1 | RNMTL1 | COPS7A |
| POMP | TERF2 | CARM1 | VPS53 | COX15 |
| FAM210A | RPS6KA4 | SKP1 | ZZZ3 | COX7A2L |
| MT-CO2 | SLC7A6OS | TYMS | CDK4 | CPEB1 |
| KIF2C | SYF2 | COX6A1 | TAF1C | CPSF2 |
| MCRS1 | MRPL32 | CCNI | PFKP | CSNK2A2 |
| STUB1 | ZNF622 | DENR | EIF3H | CTCF |
| STX16 | NARS | TBC1D15 | CFDP1 | CTNNAL1 |
| HADHB | DPY30 | RPL39L | RGPD1 | CWC27 |
| HNRNPA1 | DCP2 | CDC37 | POLR1A | CYCS |
| MGA | HAT1 | EXOC3 | BZW2 | CYTH1 |
| CCNC | SLU7 | ILF3 | SIRT1 | DARS |
| SPAG9 | SMC4 | ACTA1 | CDC14A | DARS2 |
| C11orf31 | TMEM30A | ANAPC11 | ASXL1 | DCPS |
| DDX54 | DCTPP1 | ENOPH1 | TOMM7 | DCUN1D5 |
| HSPH1 | NTHL1 | MRPL55 | C17orf49 | DDOST |
| GART | SLIRP | ANKRD28 | UTP6 | DDX20 |
| ABT1 | SMARCC2 | TAF1B | EIF6 | DDX23 |
| APEX1 | ATAD3A | MIOS | PTBP2 | DDX24 |
| SNX1 | CAPN1 | PPP2CA | XPNPEP3 | DDX25 |
| PRPF40B | SNW1 | POLR1E | SUZ12 | DENND6A |
| NUSAP1 | MRPL49 | NDUFS8 | SFR1 | DERL1 |
| CCNB1 | STAG2 | CDK16 | CSNK1D | DHX35 |
| TIMM44 | MRPS10 | POLR2K | MAP4K3 | DHX8 |
| RFC3 | TTK | ATPIF1 | XPOT | DHX9 |
| YBX1 | BRMS1L | TXNL4A | HERC5 | DICER1 |
| HADHA | DHX32 | HAUS1 | POLDIP3 | DIMT1 |
| MRPL16 | DCTN3 | ZRANB2 | TMEM5 | DLD |
| FTSJ2 | CHMP5 | TOMM5 | TXNDC17 | DLGAP5 |
| CNOT4 | GBF1 | PDCD11 | RCN2 | DLST |
| LUC7L | DLAT | SURF6 | COX5A | DNAJB14 |
| HNRNPUL1 | PELP1 | VBP1 | RNASEH2A | DNAJC2 |
| PCMT1 | UBA5 | TTF1 | PPP2R2A | DOHH |
| CCDC59 | DNAJA1 | EIF3J | SIN3A | DPH1 |
| SUMO1 | FKBP1A | CDC25C | PIK3CD | DRG2 |
| MRPL53 | GOLPH3 | UBE2K | HDAC9 | DTYMK |
| PREPL | GEMIN7 | ALDH18A1 | CDC25A | DUS4L |
| MRPL18 | MRPL14 | FBXW5 | ZC3H18 | DUSP12 |
| MTHFD1L | MEMO1 | DIS3 | RPS29 | DYNC1I1 |
| CNOT7 | TRAPPC11 | SRSF11 | PRPS1 | DYNC2H1 |
| NEDD4 | POLA1 | HNRNPD | CCT5 | E2F4 |
| IPO7 | H3F3B | EXOSC4 | NDUFS4 | EED |
| SNRPC | CCDC86 | GLS | NXF2B | EFTUD1 |
| ATXN7 | ATG12 | IQCB1 | C19orf53 | EIF2B2 |
| ATP5O | PJA2 | ECD | NDUFAF4 | EIF3CL |
| TAF4 | UBE2G1 | CPSF6 | MSH6 | EIF3K |
| ATM | ACTL6A | NAA38 | SMARCC1 | EIF4A1 |
| PPM1B | MOB4 | EIF5A2 | MSN | EIF4E |
| BTAF1 | RACGAP1 | ORMDL2 | LAS1L | EIF4EBP1 |
| LAMP2 | PCBP1 | LRRC40 | C17orf75 | EIF4G3 |
| PRRC1 | PPP1R12A | PSME3 | NIT2 | EIF4H |
| NDUFA1 | ASNSD1 | BRCC3 | GPX4 | EIF5A |
| VPS41 | LSM3 | ING3 | BRIP1 | EMC6 |
| TIAL1 | TUBB | EXOC2 | MPHOSPH9 | EPB41L2 |
| GEMIN5 | SET | DST | NUS1 | ERCC8 |
| TMA16 | DRG1 | RAD51 | MTERF | ETNK1 |
| SEC24A | PMS1 | UBA2 | NCAPG2 | EXOC4 |
| FBXW7 | ECHS1 | ARF1 | NGDN | EXOC5 |
| NOP58 | LARP7 | MRPS2 | SERINC1 | FAM96B |
| ATAD2B | TUBG1 | PRKDC | MRFAP1L1 | FANCI |
| IPO13 | NDUFV1 | URB2 | MORF4L1 | FANCL |
| CHD3 | C4orf27 | MRPL38 | SNRPF | FASTKD1 |
| SF1 | ZNF22 | MFAP1 | PTRH2 | FNBP4 |
| SOD2 | HIST1H4H | PPP1R8 | ACSL3 | FXR2 |
| HNRNPF | NDUFB5 | VAPA | DSCC1 | FYTTD1 |
| RPL39 | MDH1 | SRP14 | PBRM1 | FZR1 |
| SAC3D1 | NUDCD2 | UBE4A | TRMT11 | G2E3 |
| VCL | FAM60A | RSRC2 | DHX29 | GARS |
| ATP5G1 | CCNB2 | PDCD5 | SYNCRIP | GBAS |
| EIF2B3 | CPSF7 | CREBBP | SRA1 | GDAP2 |
| ERGIC2 | VPS4A | RPF2 | KHDRBS1 | GGCT |
| NDUFC1 | NUP214 | BAG2 | MYO1D | GLRX2 |
| GTF3C2 | MAPRE3 | PTCD2 | NEDD1 | GMFB |
| HSP90AB1 | RPL41 | ERCC3 | COMMD8 | GNAI3 |
| RAD50 | NDUFAF5 | FBXO25 | SLC25A3 | GNB2L1 |
| ABCE1 | ZWILCH | TMSB10 | NSA2 | GNL2 |
| PRIM1 | EMC4 | RPS10 | CALM1 | GOLGA4 |
| KDM1A | KIAA1429 | TOP2A | ATOX1 | GOLGA5 |
| TAF11 | NME1-NME2 | SLC25A11 | MTR | GOLT1B |
| ADRM1 | PARK2 | IARS2 | GABARAPL2 | GPBP1 |
| TSNARE1 | NIFK | BOP1 | EBAG9 | GRSF1 |
| BMS1 | RPS4Y1 | FBXW11 | GLOD4 | GTF2F1 |
| PPARGC1A | RTCB | IPO5 | GTF2E2 | GTF2I |
| CWC22 | PSMC5 | RAB5A | BARD1 | GTF3C1 |
| EIF4G2 | SNRPB | HIP1R | ACLY | GTPBP8 |
| HCFC1 | CDK5 | RPL9 | WDR83OS | H2AFZ |
| USP16 | PRKAR1A | SLC25A4 | ANXA1 | HARS |
| HIST2H2AA4 | SSSCA1 | OLA1 | SNRNP40 | HARS2 |
| NUP155 | HIST1H4A | SACM1L | WWOX | HDAC10 |
| DHX34 | RPL23 | JTB | EXOSC1 | HDAC5 |
| ANLN | BNIP2 | VPS33A | MED6 | HDAC7 |
| PACS1 | XRN1 | ILF2 | GLMN | HDGF |
| ZNF330 | ICT1 | CETN3 | COX16 | HDLBP |
| XPO5 | ESD | H2AFV | DHX37 | HIP1 |
| RGPD8 | RASA1 | SEC16A | NFYB | HMGCS2 |
| SLC30A5 | POLR2L | AIFM1 | PAIP2 | HNRNPA2B1 |
| DNAJC5G | HIST1H4K | KPNA3 | GSTO1 | HNRNPH1 |
| MICU2 | HIST1H2BL | RRP1B | EP300 | HNRNPL |
| NUP85 | ZNF638 | PLEC | H2AFX | HSDL2 |
| TRNT1 | C2orf47 | HAUS3 | STRAP | HSPA14 |
| PRC1 | MLLT3 | SAAL1 | NTPCR | IKBKAP |
| PPIL4 | NDUFB10 | TAF6L | API5 | IMPA1 |
| PPIF | C11orf58 | TRIP13 | G3BP1 | IMPDH2 |
| COPE | M6PR | AGPAT5 | PGAM1 | INSR |
| POLR1D | NARG2 | PPP2R5C | AP1S1 | IPO11 |
| ZNF830 | PUSL1 | HNRNPA1L2 | POT1 | IPO4 |
| METTL10 | COX19 | NUDT5 | RBM12 | IPO9 |
| SUPT3H | KDELR1 | BECN1 | HUS1 | IQGAP1 |
| PEBP1 | NOC4L | WDR82 | ACSS2 | ISY1 |
| CHEK2 | INTS7 | DYNLL1 | SMNDC1 | KANSL2 |
| SLC25A6 | FAM216A | ZNF593 | GUF1 | KAT2A |
| SNX17 | RGPD3 | HDAC6 | YARS | KHSRP |
| MT-ND1 | EDRF1 | WIBG | POLR3H | KIAA0368 |
| BCLAF1 | HMGN1 | RHEB | GTPBP10 | KIAA1033 |
| OTUD6B | POP4 | AP2A1 | DNAJC7 | KIF14 |
| RRAGA | CCNH | MYEF2 | CKAP5 | KIF18B |
| LTN1 | RBM4 | SUCLG1 | SSNA1 | KIF1A |
| DIDO1 | BCCIP | CTDSPL2 | MRPL48 | KIF2A |
| OSTC | MRPL20 | PMPCA | RRS1 | KRAS |
| ATG7 | HIST1H4L | TARS | TXNDC9 | LACTB2 |
| PAXBP1 | UQCRQ | CDK3 | NSUN2 | LAMTOR2 |
| CARS | NSF | SUGT1 | RPP40 | LAMTOR3 |
| NOL11 | EIF1AY | KAT8 | EIF5 | LAMTOR5 |
| RABEPK | TRUB2 | SNX5 | EIF3M | LAPTM4A |
| H1FX | ZNHIT3 | GEMIN2 | UBE2D2 | LDHB |
| PPIB | TRUB1 | YEATS4 | SRSF4 | LEMD3 |
| CLK1 | ATP5H | RFC4 | RBM22 | LMNA |
| RPS27L | UQCR10 | SMC1A | LARP4B | LMNB2 |
| EHD1 | MAGOH | MCM4 | DAZAP1 | LONP1 |
| MYO5A | SEC63 | VTI1B | ASNA1 | LRPPRC |
| MRPL11 | GPN1 | LRRC47 | AP1G2 | LSG1 |
| CCT2 | HSPA2 | COIL | LTA4H | LSM1 |
| MT-ATP6 | GLE1 | POLR3F | FASN | LSM10 |
| VPS39 | PIN1 | USMG5 | FIS1 | LSM5 |
| PIAS2 | RAB11B | BRD8 | RPL22 | LSM7 |
| TCP1 | RRP8 | NDUFV2 | DDX50 | LSMD1 |
| RIOK2 | CIRH1A | C21orf2 | PSMD2 | MACF1 |
| VPS16 | RAD18 | URI1 | GPS1 | MAK16 |
| VTA1 | FANCD2 | GOLGB1 | RIOK1 | MAP1S |
| MTFMT | BYSL | MRPL21 | PCID2 | MAP2K2 |
| CHD1 | DDX27 | CDC23 | TBCB | MAPK1IP1L |
| CDC20 | TOPORS | TRIM28 | CKS1B | MCM2 |
| FBXO5 | EP400 | SIRT7 | KDM3A | MCM6 |
| NDUFB3 | SMARCA1 | ZWINT | MAP4 | MED21 |
| NMD3 | HNRNPA3 | CTBP1 | IMMT | MED23 |
| ETAA1 | ZCCHC3 | GTF2H1 | ZUFSP | METAP1 |
| YWHAG | CASP3 | DNAJC9 | WRN | METTL13 |
| OSBPL8 | THAP2 | RPS27 | DHX36 | METTL18 |
| LSM2 | RPL28 | MTA2 | SLC25A32 | METTL5 |
| SMARCA2 | COX7A2 | KIF5B | HTT | MFN2 |
| ECT2 | TP53BP1 | PYGB | NCOR1 | MLF2 |
| DNAJC13 | CHCHD4 | DHX38 | FIP1L1 | MLLT1 |
| PBDC1 | DNHD1 | KAT2B | DCTN6 | MMADHC |
| NOC2L | MRPL37 | DNAJA3 | RAP2B | MNAT1 |
| ACP1 | PDIA4 | RAC1 | ZNF644 | MOV10 |
| PLEK | MRPS34 | NDUFB4 | EIF2A | MPND |
| MBD2 | BUB1 | WDR74 | HNRNPH3 | MRE11A |
| UBE2C | SSBP1 | UQCC2 | TPX2 | MRPL13 |
| MED7 | SEC62 | SRSF9 | TADA2B | MRPL24 |
| EDC4 | RPS2 | SNX2 | KIF4A | MRPL34 |
| ZCCHC9 | DPM1 | PRPF39 | ECHDC1 | MRPL43 |
| OASL | SMN1 | CHD8 | ARF5 | MRPL46 |
| ATP5E | INS-IGF2 | NDUFAF6 | GNPAT | MRPL50 |
| MRPL10 | PTTG1 | NAA11 | RPS6KB1 | MRPL51 |
| HIST2H2AA3 | NCOA1 | SRRM2 | ELAC2 | MRPL54 |
| ARPP19 | SMU1 | BOLA3 | RALY | MRPS11 |
| SNRNP25 | GLTSCR2 | EEA1 | NOP16 | MRPS12 |
| TRA2B | ACTB | VPS28 | POLR2J | MRPS14 |
| SUMO2 | SENP6 | STX4 | PUM1 | MRPS15 |
| CAP1 | MPHOSPH10 | MYH9 | GFM2 | MRPS17 |
| BPTF | PPRC1 | MYO1B | CHAMP1 | MRPS28 |
| NUCKS1 | CENPQ | PCBP3 | FMR1 | MRPS35 |
| SMARCA5 | SHMT2 | NUDC | IMP4 | MRRF |
| LTV1 | SYT1 | SERBP1 | DNAJC5B | MRTO4 |
| SIRT5 | VPS54 | CPSF3 | TMPO | MTA1 |
| DTWD1 | FAM175B | FAM98B | SLC30A9 | MTBP |
| WDR46 | ATG5 | ANAPC1 | MAP2 | MTERFD2 |
| CNPY2 | HIBCH | DNAJA2 | SHOC2 | MTERFD3 |
| STAG1 | FEN1 | SMC5 | RTCA | MTIF2 |
| FXR1 | CUL1 | DHDDS | E2F6 | MYC |
| UBE3A | VDAC2 | STOML2 | FUBP1 | MYO1C |
| MRPL27 | FOXO3 | WDR5 | FARSA | NAE1 |
| RCL1 | CCNA1 | UQCRB | UGGT1 | NARS2 |
| DONSON | HIST1H4C | EXOSC3 | OCIAD1 | NBR1 |
| CTNNB1 | NOP10 | ZCCHC8 | LRR1 | NCAPD2 |
| ARPC3 | INO80C | DNAJC8 | RPAP3 | NCBP2 |
| CDK9 | RANBP1 | NSRP1 | RSL1D1 | NDRG1 |
| DNAJC19 | RNASEH1 | YWHAQ | RRP12 | NDUFA11 |
| MT-CO1 | PRPSAP2 | BCS1L | RBM42 | NDUFA3 |
| GTF2H4 | TPRKB | GTF2E1 | CKB | NDUFA8 |
| SYNE2 | SNRPA1 | SPDL1 | MIS12 | NDUFA9 |
| NOL7 | GGNBP2 | CCAR2 | RBM28 | NDUFAB1 |
| CDC25B | MRPS18A | RER1 | TFDP1 | NDUFAF2 |
| ORC2 | COX14 | NR2C1 | HELZ | NDUFAF3 |
| MRPS33 | BIRC6 | ATP5L | TMED9 | NDUFB8 |
| PLRG1 | HIST1H4B | HMGA1 | REV1 | NDUFB9 |
| ERCC6L | EPS15 | BRE | TUBB4B | NDUFC2 |
| MCTS1 | DNMT1 | TERT | IK | NDUFS2 |
| CCDC91 | CSNK2A1 | TCEB2 | TSFM | NDUFS3 |
| AASDHPPT | MRPS24 | SYMPK | MPHOSPH6 | NDUFS5 |
| SKP2 | PSMB6 | TM9SF2 | EEF1A1 | NMT1 |
| MIS18A | SMG9 | GTF2A1 | MAP2K4 | NOA1 |
| TPM1 | ERP29 | POLE | TEFM | NONO |
| ACAT1 | CYB5B | TAF5 | ELP3 | NOP2 |
| PHAX | GOSR2 | POLR2G | SKIV2L2 | NOP9 |
| KNCN | PIGW | EPB41L3 | TTLL12 | NRAS |
| GMNN | NR3C1 | FNTA | ATF7IP | NSMCE2 |
| TRMT112 | SUCLA2 | RPL26L1 | LRWD1 | NUF2 |
| HSPA1B | C10orf11 | HACL1 | DHX57 | NUP160 |
| RAB33B | PNRC2 | RAB21 | DDX5 | NXT2 |
| PTGES3 | ALG5 | MTERFD1 | TOMM20 | OGT |
| HDAC3 | ENDOG | CYC1 | ASF1A | OPA1 |
| WAC | MRPL19 | GADD45A | ARF6 | ORC4 |
| NSUN4 | MYL6 | MYCBP2 | KLC1 | ORC5 |
| DRAP1 | DDX42 | PDS5A | TDG | PABPC4 |
| RAB1B | SEC11C | EIF2AK2 | PARN | PAFAH1B1 |
| PSMA7 | RABAC1 | SKIL | KPNA1 | PAICS |
| SH3GL2 | MRPL47 | NDUFA4 | ANP32B | PAK2 |
| C6orf211 | TBL3 | COX6C | HDAC1 | PALB2 |
| WDR43 | SAR1A | MEPCE | ORC3 | PAPD4 |
| NFATC2IP | STAU1 | TADA2A | HSBP1 | PCBP2 |
| SLC25A46 | EXOSC8 | IBTK | PTBP1 | PCF11 |
| TFB1M | CCNK | ATRAID | UBQLN1 | PCNP |
| HSPA5 | MTHFD1 | MCM5 | THOC3 | PDCD6IP |
| MRPL22 | COA7 | GTF3C6 | PFDN1 | PDE12 |
| DNAJB9 | H3F3C | UCHL1 | CCAR1 | PDHB |
| STX3 | SMARCD2 | PPIA | NUPL2 | PET100 |
| RING1 | NME1 | PIK3C3 | CAPRIN1 | PFDN5 |
| SMARCB1 | EAPP | DDX19A | DDX6 | PHF20 |
| MRGBP | NCAPH | POLR3G | SEC24C | PIAS4 |
| CTPS1 | TSN | FAM207A | MTF2 | PLK1 |
| FBL | TACO1 | RPL22L1 | RWDD1 | PLK2 |
| BTRC | MKKS | AP2M1 | FRYL | PLS3 |
| PPIL3 | MTPAP | ITGB3BP | PSMB11 | PMPCB |
| DHX15 | NOP56 | SEC31A | NHP2 | PNPT1 |
| CENPJ | SRP54 | MTDH | CMC2 | POLE3 |
| AP3M1 | AURKAIP1 | IWS1 | PRELID1 | POLG |
| TAF9 | UBL5 | DDX17 | MDN1 | POLR2A |
| PFDN6 | PARVA | ACTR1A | TARBP1 | POLR2B |
| NDUFAF1 | TRIP12 | UTP14A | PHF3 | POLR2H |
| MT-ND5 | CEP290 | UBE2N | HSP90B1 | POLR2J3 |
| FKBP1B | CDKN2AIP | MAGOHB | EIF4B | POLR3B |
| VPS37A | UBA3 | ATF4 | SMC6 | POLR3C |
| GGA3 | BAZ2A | ACBD3 | BAG5 | POLR3E |
| MCM3 | VDAC1 | TOMM22 | TMED5 | PPA2 |
| USP8 | UFD1L | EIF2B5 | CHMP2B | PPAT |
| PPP1CB | GSTA3 | ATF1 | DHX33 | PPIG |
| AP1B1 | AATF | TJP2 | TIMM22 | PPM1A |
| PDS5B | TAF15 | UTP15 | PYURF | PPP2R5E |
| NCAPG | PFAS | TGOLN2 | DDX55 | PPP3CA |
| EXOSC5 | PPP2R4 | INTS3 | EIF4G1 | PPWD1 |
| STIP1 | COX7C | NDUFA12 | IARS | PRCC |
| OIP5 | MRPL40 | USP24 | RRM2B | PRMT6 |
| DDT | TTC37 | TIMM23 | TOP2B | PRPF38B |
| HIST2H4A | MYBBP1A | SEC23B | CDS1 | PRPF4 |
| NDUFA7 | P4HB | RRM2 | NCBP1 | PRRC2C |
| MT-ND4 | RPS23 | TAF1A | PTCD3 | PSMA5 |
| HERC2 | TMEM14C | AIMP2 | TM9SF3 | PSMC3 |
| DDX21 | ATAD1 | MARS | SMURF2 | PSMC6 |
| HBS1L | TRAPPC8 | RPL27A | ZCCHC17 | PSMD13 |
| CLPX | AP2B1 | DGUOK | PDCL3 | PSMD5 |
| CTR9 | UBL4A | POP5 | MTOR | PSME4 |
| MDH2 | SNRPB2 | BTF3 | RAD21 | PTCD1 |
| MIF | PCM1 | PSMB4 | MITD1 | PUM2 |
| NUP43 | ZFR | ACTN1 | PEX14 | PUS7 |
| IER3IP1 | ATP5G3 | ARHGDIA | CDC123 | PYGL |
| MRPS22 | MRPL45 | RNASEH2B | TGS1 | RAB5C |
| ZC3H8 | NSFL1C | PARP1 | GSPT1 | RAB7A |
| HIST2H2AC | CUTA | MRPL9 | MYH14 | RAB8A |
| SAP18 | DDX39B | DNM1L | LYPLA1 | RAD23B |
| SACS | GFM1 | RRP9 | NXF2 | RAD51AP1 |
| LUC7L3 | PHIP | CCZ1B | DNTTIP2 | RANBP3 |
| BUB3 | UTP18 | MRPS23 | ACIN1 | RAP1A |
| KPNA2 | HIST1H4E | KNTC1 | PSMD12 | RBBP6 |
| MTMR6 | SMN2 | PRDX3 | GCSH | RBM15 |
| SLMO2 | PRPF6 | RAB1A | HDAC2 | RBM39 |
| RAB11A | ILK | CTNNA1 | MRPL44 | RBM6 |
| MAPT | PPP1CC | GRPEL2 | FKBP4 | RBM8A |
| SUMO4 | NOLC1 | AP1G1 | ENY2 | RCC2 |
| EBNA1BP2 | UBE2S | VPS36 | WDR18 | RECQL |
| CMPK1 | SEC13 | GOSR1 | TOMM70A | RFC2 |
| ACTG1 | MYH10 | RPL36A | NUP50 | RHOA |
| CDK5RAP3 | FUBP3 | SMARCE1 | MRPS5 | RIF1 |
| ATF2 | ATAD3B | CCNA2 | DDX1 | RINT1 |
| TPD52L2 | RPRD1A | RB1CC1 | DNAJB1 | RNF2 |
| GTF3C3 | FOXO1 | USP11 | HTATSF1 | RNH1 |
| MRS2 | BDP1 | RRAGB | NDUFB7 | RNPEP |
| HECTD1 | SRPK1 | COA3 | KLHDC2 | RPN1 |
| NLE1 | EXOSC2 | CSNK1A1 | COPS5 | RPP38 |
| GTF3A | GDI1 | DNAJC6 | EIF3E | RPS12 |
| DSP | YTHDC2 | COX5B | STX18 | RPS16 |
| NAP1L4 | RAP1B | INTS6 | TUBA4A | RPS17L |
| ARFIP1 | SNAP23 | SDHA | DEPDC7 | RPUSD4 |
| TUBA3C | GPN3 | FSCN1 | YTHDF2 | RSF1 |
| VPS51 | TMEM258 | POP7 | AP3D1 | SART3 |
| CDC42 | NIPBL | MTHFD2 | RPL36AL | SBDS |
| ANKRD52 | SPAG5 | GIGYF2 | GTF2H3 | SCO2 |
| VPS52 | PSMG1 | WDR25 | NOL9 | SCYL2 |
| NAP1L1 | PPP1CA | SRP9 | POLE2 | SDHB |
| LLPH | UFM1 | EIF4E2 | MRPL35 | SEC11A |
| MRPL28 | NUP107 | CASC3 | SNUPN | SEC61A1 |
| ESCO1 | MRPS18B | PRKRA | NUP88 | SEC61G |
| CALM3 | MTRF1 | VPS25 | CDC16 | SENP1 |
| RPL8 | YEATS2 | C11orf48 | RRP7A | SENP2 |
| THOC7 | NUFIP1 | FASTKD2 | DYNC1LI1 | SENP3 |
| RDX | MTG1 | TFAM | PSMA4 | SENP5 |
| GAPVD1 | HDAC11 | RQCD1 | RBX1 | SEPHS1 |
| RFC5 | PUS1 | FBXO28 | TNPO2 | SF3A3 |
| PEX3 | HSPA4 | UBE2M | E2F1 | SIN3B |
| RELA | PDHA1 | PWP2 | RAB40AL | SIRT3 |
| ARL1 | AP2A2 | SRP68 | CHCHD1 | SIRT6 |
| ACOT13 | MDC1 | HSD17B10 | PRMT5 | SIVA1 |
| SART1 | COG8 | UCHL3 | NUDCD1 | SLC25A13 |
| LMNB1 | TBP | CDC6 | FASTKD3 | SLC25A5 |
| RSL24D1 | RPL3L | DDX56 | SND1 | SLC36A4 |
| RAE1 | SRP19 | VPS4B | ANAPC2 | SMARCAD1 |
| PSMB7 | GSTA1 | RPL7L1 | ST13 | SMC2 |
| SYAP1 | TTC27 | PSMD14 | FAM208B | SMEK2 |
| TIAM1 | URM1 | PFDN4 | RAD51C | SMG1 |
| PPIL1 | GLO1 | EIF3L | DNAAF2 | SMG6 |
| ARHGEF1 | CENPU | TAF5L | TAF2 | SMG7 |
| LEO1 | GTF2A2 | KRI1 | TUBGCP3 | SNIP1 |
| RNMT | TXNL1 | USP37 | FKBP1C | SNX4 |
| FAM136A | ANAPC4 | AHNAK | PDIA6 | SOGA1 |
| PSMG3 | PPA1 | RAD52 | SHC1 | SPEN |
| ESRRA | TIA1 | CISD1 | COQ3 | SPTBN1 |
| MAPK6 | PES1 | STAMBP | SEH1L | SQSTM1 |
| SNRPE | SRRM1 | MRPS27 | PARK7 | SREBF1 |
| ATRX | RGPD4 | OXA1L | NDUFA2 | SREK1 |
| XPO4 | REEP5 | CUL2 | SRSF5 | SRP72 |
| RBBP5 | MAX | TGDS | RBM25 | SRPK2 |
| WDR89 | TBL1XR1 | LARS2 | TRAPPC4 | SRPR |
| VPS26A | CCDC47 | AMD1 | SOS1 | SRSF6 |
| PRDX1 | PRICKLE4 | DDX31 | CLTB | SSB |
| TRMT61B | HMGB2 | MRPL42 | NAT10 | SSR2 |
| CS | TMED7 | SMARCA4 | HSPA8 | SSR4 |
| SNX9 | GCN1L1 | FUS | ATP5D | SSRP1 |
| HSPA1A | RRAS2 | SURF1 | HMGCR | ST14 |
| RSRC1 | MED4 | MSH2 | C14orf2 | STAM |
| SP2 | GTF2H2 | RNF4 | GEMIN4 | STX1A |
| USP47 | TAF10 | HLTF | SPC25 | STX6 |
| RANBP2 | WBP4 | PFN1 | ZCCHC6 | STX7 |
| RPAP1 | TVP23B | PIGF | UBAP2L | STXBP3 |
| RBBP7 | AARS | ARL14EP | FLNC | SWT1 |
| ATP6V1A | PDCD2 | ZC3H14 | USP10 | SYS1 |
| SOD1 | DHX16 | PUF60 | C1QBP | TAF1 |
| SNRPD3 | PPP2R1B | AURKA | TRIM37 | TAF13 |
| UQCRH | NEK2 | PCGF6 | GADD45GIP1 | TAF1D |
| KRR1 | PPP2R3C | CDK19 | EXO1 | TAF7 |
| CNOT8 | MTX2 | BUB1B | COX6B1 | TATDN1 |
| ZC3H11A | ATXN1 | GANAB | FH | TAX1BP1 |
| MYO5B | VAMP2 | FTH1 | ERCC5 | TBCA |
| UBA52 | ESF1 | UPF2 | AHCTF1 | TBPL1 |
| AIMP1 | HINT1 | TSG101 | DNPEP | TCEB1 |
| UPF3B | PAK1IP1 | HUWE1 | SMG8 | TERF1 |
| DNAJC17 | GSTA2 | SNAP29 | DBF4 | TEX10 |
| HIST1H2AG | PSMD3 | PML | CALU | TFRC |
| GPKOW | AAMP | SUPT16H | ZSWIM8 | THUMPD1 |
| BLM | MRPS7 | CUEDC2 | NXF5 | TIMM13 |
| DPH2 | C7orf55-LUC7L2 | YWHAZ | OAZ1 | TIMM17A |
| IDE | TOPBP1 | NAA20 | LANCL1 | TIMM50 |
| DUSP2 | CENPF | SCO1 | ATIC | TIPRL |
| C8orf59 | THUMPD3 | YKT6 | MRPL36 | TMA7 |
| HIST1H4I | LARP4 | FKBP3 | PSMD8 | TMCO1 |
| MCUR1 | COX11 | SUPT4H1 | TMEM66 | TMED10 |
| COPB2 | TAF9B | GNB1 | DCAF13 | TMEM126A |
| POLI | LMAN1 | TIMM8A | CMSS1 | TMEM208 |
| MRP63 | GFPT1 | DDX3X | PCNT | TMX1 |
| NIF3L1 | NFX1 | UBE2G2 | AKAP9 | TMX2 |
| CDK7 | MBTPS2 | BORA | UPF3A | TNKS1BP1 |
| CDK8 | ZCCHC10 | REXO4 | GTF2B | TOP1 |
| CDS2 | PSMD4 | CTDP1 | INTS2 | TP53 |
| ACAT2 | TCERG1 | TIMM17B | ASF1B | TPI1 |
| SS18L2 | RB1 | ACADM | ARFGEF1 | TPR |
| SCFD1 | NECAP1 | CAPN7 | RANGAP1 | TRAM1 |
| COX8A | SCP2 | TOX4 | UBE2Q2 | TRIT1 |
| DMAP1 | CLASP1 | SEC23A | SRRT | TRMT10C |
| GABPA | TIPIN | RRP15 | DUT | TRMU |
| PSMA8 | PRR14 | RPL15 | ATXN10 | TROVE2 |
| ESR1 | GTF2F2 | SUV39H1 | SCAF11 | TRRAP |
| TRMT6 | MRPL33 | MRPS16 | EXOC1 | TSEN15 |
| NUTF2 | MRPL12 | H3F3A | PBK | TSR1 |
| CUL4B | HELLS | RPS19BP1 | UBC | TUBA1A |
| CNOT2 | KIF23 | RPA3 | PRKACA | TUBA1C |
| PATL1 | MAPRE2 | DAD1 | FARSB | TUBB4A |
| MVP | ADNP | VAMP8 | PRKRIR | TUFM |
| RAB18 | CLINT1 | POGZ | DUS1L | TXN |
| HSPB1 | YME1L1 | WDR33 | PPP2CB | UAP1 |
| KAT5 | CENPA | DOCK7 | CAT | UBA6 |
| ATP5J | GOLGA2 | CCNE1 | CDC34 | UBB |
| PARP2 | NUP188 | CSTF2 | EMC2 | UBE2D3 |
| MT-CYB | GSTA4 | PPP6R3 | MRPS18C | UBE2H |
| HSPD1 | MRPL4 | RCC1 | GDI2 | UBE2T |
| PLK3 | NOL6 | COQ5 | CCDC14 | UBR4 |
| NDUFB2 | TPT1 | HNRNPDL | RBBP4 | UBR7 |
| CNTLN | RPLP1 | LRTOMT | PSMD10 | UBTF |
| PPID | ARPC4 | MEA1 | PDHX | UBXN4 |
| KANSL1 | SUB1 | ADSL | HNRNPAB | UCHL5 |
| BBS10 | INTS8 | PQBP1 | TRMT13 | UFC1 |
| EIF1B | TTC1 | CKAP2 | USP7 | UQCRC1 |
| RBM5 | TDP2 | EEF1B2 | ITCH | UQCRC2 |
| MTIF3 | PRDX4 | VAMP7 | RBMX | USE1 |
| GSPT2 | TTC4 | PAXIP1 | POLR2C | USP34 |
| ZNF318 | RPS4Y2 | CRNKL1 | CUL4A | UTP11L |
| RPP21 | SMARCD1 | POP1 | PRDX2 | UTP20 |
| BANF1 | EP400NL | CSTF2T | SSR1 | UTP3 |
| ARL8B | EIF4A2 | PPP4R1 | FOPNL | VAMP4 |
| IPO8 | ATP5B | VPS35 | RRN3 | WBSCR27 |
| RPL21 | TAF6 | ARID1A | EIF1 | WDHD1 |
| BRMS1 | DNAJC10 | VEZT | HDDC2 | WDR3 |
| SKIV2L | XIAP | USP1 | DHX40 | WDR31 |
| NAA50 | EIF2S3 | RBM3 | SPTLC1 | WDR6 |
| MRPL3 | EZH2 | PNN | NAA35 | WDR77 |
| RAB10 | NME2 | POLR3D | SEC22A | YTHDC1 |
| LARP1B | SPAST | MRPL2 | ARMC1 | YWHAB |
| ZMYM2 | TCEA2 | NPEPPS | PRPF4B | YWHAE |
| KIFC1 | ACTR3 | NDC80 | SREBF2 | ZBTB11 |
| FAM96A | VRK1 | USP9X | LIPT1 | ZDHHC6 |
| CDK2 | NIP7 | METTL3 | HSF2 | ZFAND1 |
| HIST2H2BE | C6orf203 | RFC1 | SRSF2 | ZMAT2 |
| DIS3L |  |  |  |  |
